# Hypoxia-Induced miR-210 Is Necessary for Vascular Regeneration upon Acute Limb Ischemia

**DOI:** 10.3390/ijms21010129

**Published:** 2019-12-24

**Authors:** Germana Zaccagnini, Biagina Maimone, Paola Fuschi, Marialucia Longo, Daniel Da Silva, Matteo Carrara, Christine Voellenkle, Laura Perani, Antonio Esposito, Carlo Gaetano, Fabio Martelli

**Affiliations:** 1 Laboratory of Molecular Cardiology, IRCCS Policlinico San Donato, 20097 San Donato Milanese, 20097 Milan, Italy; maimone.biagina@gmail.com (B.M.); paola.fuschi@grupposandonato.it (P.F.); marialucia.longo@grupposandonato.it (M.L.); danielsilvars@ymail.com (D.D.S.); carrara.matt@gmail.com (M.C.);; 2 Preclinical Imaging Facility, Experimental Imaging Center, San Raffaele Scientific Institute, 20132 Milan, Italy; perani.laura@hsr.it (L.P.); esposito.antonio@unisr.it (A.E.); 3 Vita-Salute San Raffaele University, 20132 Milan, Italy; 4 Laboratorio di Epigenetica, Istituti Clinici Scientifici Maugeri, via Maugeri 4, 27100 Pavia, Italy; carlo.gaetano@icsmaugeri.it

**Keywords:** miR-210, angiogenesis, limb ischemia, myocardial infarction

## Abstract

Critical limb ischemia is the most serious form of peripheral artery disease, characterized by severe functional consequences, difficult clinical management and reduced life expectancy. The goal of this study was to investigate the miR-210 role in the neo-angiogenic response after acute limb ischemia. Complementary approaches were used in a mouse model of hindlimb ischemia: miR-210 loss-of-function was obtained by administration of LNA-oligonucleotides anti-miR-210; for miR-210 gain-of-function, a doxycycline-inducible miR-210 transgenic mouse was used. We tested miR-210 ability to stimulate vascular regeneration following ischemia. We found that miR-210 was necessary and sufficient to stimulate blood perfusion recovery, as well as arteriolar and capillary density increase, in the ischemic muscle. To clarify the molecular events underpinning miR-210 pro-angiogenic action, the transcriptomic changes in ischemic muscles upon miR-210 blocking were analyzed. We found that miR-210 impacted the transcriptome significantly, regulating pathways and functions linked to vascular regeneration. In agreement with a pro-angiogenic role, miR-210 also improved cardiac function and left ventricular remodeling after myocardial infarction. Moreover, miR-210 blocking decreased capillary density in a Matrigel plug assay, indicating that miR-210 is necessary for angiogenesis independently of ischemia. Collectively, these data indicate that miR-210 plays a pivotal role in promoting vascular regeneration.

## 1. Introduction

Peripheral artery disease (PAD) is a pathological condition in which blood flow is insufficient to meet metabolic demand. This pathology is estimated to affect more than 200 million people worldwide, with a high incidence not only in high-income countries, but also in low- and middle-income areas of the world [1,2]. PAD is mostly caused by stenosis, embolism, or thrombosis involving the arteries supplying the leg [3]. Critical limb ischemia is the most serious form of PAD, characterized by severe functional consequences, difficult clinical management and reduced life expectancy [4].

A crucial component of ischemia is hypoxia that triggers a complex program of responses aimed at counteracting the effects of decreased oxygen tension, removing irreversibly damaged cells, restoring tissue perfusion, and regenerating the vascular network [5]. A specific subset of miRNAs, named hypoxia-induced miRNAs or hypoxamiRs, is expressed in response to hypoxia and modulates several target genes involved in these processes [6]. Among these miRNAs, miR-210 can be considered the master hypoxamiR, since it has been found to be upregulated by hypoxia in all cell types and tissues tested to date, carrying out a variety of functions [7]. Indeed, miR-210 is a target of Hypoxia-Inducible Factor 1-(HIF1A) [5], which directly activates its transcription under low oxygen tension [7,8].

Preclinical and clinical evidence confirm the crucial role of miR-210 in the regulation of cell responses to hypoxia and ischemia. Indeed, miR-210 has been found upregulated in different ischemic diseases, such as ischemic wounds [9], hindlimb ischemia in mice [10], brain transient focal ischemia in rats [11], myocardial infarction in humans [12], and in heart failure of diabetic patients affected by dilated ischemic cardiomyopathy [13].

Under hypoxia, miR-210 modulates mRNA transcripts involved in multiple processes of cellular response. In particular, it can repress mitochondrial metabolism, promoting the shift from mitochondrial respiration to glycolysis [14], inhibiting apoptosis [10,15] and supporting stem-cell survival [16,17]. Accordingly, in limb ischemia, we previously found that miR-210 overexpressing transgenic mice were protected from acute ischemic damage to skeletal muscle and blood vessels [18]. Using a complementary approach, we also found that miR-210 inhibition increased acute skeletal muscle damage with a ROS-mediated mechanism due to insufficient down-regulation of oxidative metabolism upon ischemia [10].

MiR-210 seems to play a role not only in the acute response to hypoxia/ischemia, but also in the following phases when the vascular network is regenerated, re-establishing oxygen and nutrient supply [19]. Indeed, in vitro data show that miR-210 induces capillary-like structures formation and endothelial cell migration [6,15,19]. This evidence prompted studies aimed at demonstrating the ability of miR-210 to stimulate angiogenesis in ischemic disease models. Driven by a translational purpose, in these investigations, an overexpression strategy in the ischemic tissue was adopted, showing miR-210′s ability to stimulate angiogenesis in heart [20,21,22,23], brain [24,25] and limb ischemia [26]. However, a combination of gain- and loss-of-function approaches is instrumental in providing a clear picture of miR-210 implication in the pathogenetic mechanisms underpinning the ischemia-induced angiogenic response. Using complementary and technically independent approaches in parallel experiments, we found that miR-210 plays a pivotal role in promoting the vascular regeneration and angiogenesis processes following critical ischemia.

## 2. Results

### 2.1. MiR-210 Expression Is Induced during the Neo-Angiogenic Response Following Ischemia

To test the role played by miR-210 in the neo-vascularization process that follows acute hindlimb ischemia, C57BL/6N male mice underwent femoral artery dissection [27], and miR-210 expression was measured at different time points after ischemia in gastrocnemius muscles (Supplementary Figure S1). In keeping with previous findings obtained in a different mouse strain [10], miR-210 levels were significantly higher in ischemic muscles compared to non-ischemic contralateral controls, reaching the maximal upregulation 3 days after surgery and remaining high until day 7.

Next, we determined the changes in capillary density in gastrocnemius muscles at different time points after ischemia. After surgery, capillary density decreased to a minimum at 2 days after ischemia, then started to recover and returned to basal level at day 14 (Supplementary Figure S2).

Since the neo-angiogenic response largely overlapped with miR-210 induction, we wondered whether miR-210 might play a role in the vascular regeneration process that follows ischemia.

To test this hypothesis, two complementary approaches were adopted, of either gain- or loss-of-function. In the loss-of-function approach, miR-210 was blocked by systemic administration of an LNA-modified complementary oligonucleotide (ANTI-210). Control mice received a scrambled (SCR) sequence. Given that we previously demonstrated that miR-210 blockade increases acute ischemic tissue damage [10], we injected the ANTI-210 five days after surgery, i.e., after the maximal vascular damage phase (day 2), and when the regeneration phase had started (Supplementary Figure S2). Then, we evaluated the angiogenic response 2 and 9 days later (at day 7 and day 14 after surgery, respectively). The experimental plan is schematized in Supplementary Figure S3A.

In the reciprocal gain of function approach, we took advantage of a doxycycline-inducible miR-210 transgenic mouse strain (Tg210) that we had generated and validated in the past [18]. In this case, miR-210 overexpression was induced by doxycycline administration starting from day 4 after surgery and neovascularization was assayed 3 days later. To account for potential effects of the drug, doxycycline was administered to control WT mice as well (Supplementary Figure S3B).

First, we tested the effectiveness of miR-210 modulation in both models. We found that ANTI-210 efficiently blocked miR-210 expression in ischemic muscles compared to ischemic SCR controls, both at 2 and 9 days after treatment (Supplementary Figure S4A). Then, we analyzed miR-210 expression levels in the transgenic model in a time course. We observed that doxycycline administration led to rapid induction of miR-210 expression in the non-ischemic muscles of Tg210^Doxy^ compared to WT^Doxy^ controls (Supplementary Figure S4B). Of note, miR-210 expression remained within physiological limits [7,15,19].

Thus, two independent models were established to study miR-210 role in the vascular regeneration following ischemia.

### 2.2. MiR-210 Stimulates Blood Flow Recovery In Vivo after Hindlimb Ischemia

**Figure 1:**
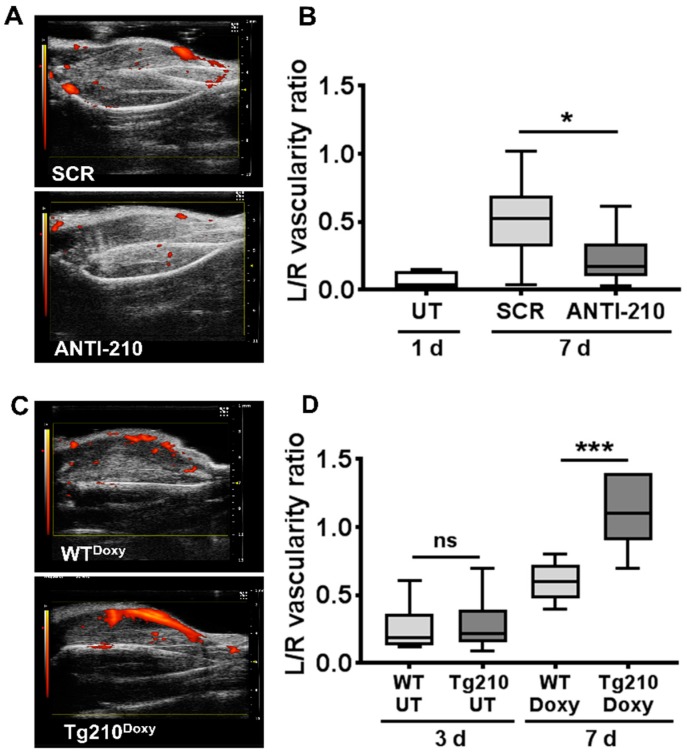
MiR-210 regulates blood flow recovery in vivo. Calf perfusion was monitored by ultrasound in power Doppler mode. Scale bar: 2 mm. (**A**,**C**) Representative images of the ischemic calf of the indicated groups taken at 7 days after ischemia; (**A**) scrambled (SCR) and ANTI-210, (**C**) Wild Type (WT)^Doxy^ and Tg210^Doxy^. (**B**,**D**) Box plots show perfusion, expressed as vascularity ratio; (**B**) untreated (UT) mice at day 1 and SCR and ANTI-210 treated mice 7 days after ischemia (*n* = 7–9); (**D**) WT and Tg210 mice, either before miR-210 induction (untreated, UT), at 3 days after surgery or treated with doxycycline (Doxy) at day 7 of ischemia (*N* = 5–6; for both panels, Anova multiple comparison * *p* < 0.04, *** *p* = 0.0008, ns = not significant).

To investigate the functional role of miR-210 in the neo-vascularization process following ischemia, we measured calf perfusion by ultrasound (Figure 1). Before miR-210 blocking, we measured the residual calf perfusion of ischemic untreated mice 1 day after surgery. As expected, perfusion, expressed as vascularity ratio, was markedly decreased in the ischemic limb (Figure 1B). Then, mice were randomized in two groups of treatment, SCR or ANTI-210. We observed a significant reduction of calf perfusion in ANTI-210 mice compared to SCR controls, 7 days after ischemia (Figure 1A,B).

Next, perfusion recovery was measured in Tg210 mice. Hindlimb ischemia was induced, and calf perfusion was assayed in both WT and Tg210 mice at 3 days of ischemia. We found no significant difference in residual perfusion between the two groups (Figure 1D), indicating that similar levels of vascular damage were induced. Following doxycycline administration, we observed a significant improvement of perfusion in Tg210^Doxy^ mice at day 7 of ischemia (Figure 1C,D).

### 2.3. MiR-210 Expression Increases Arteriolar Length Density and Capillary Density after Hindlimb Ischemia

To corroborate the blood perfusion data, morphometric analysis of the vascular system was performed in histological sections of gastrocnemius muscles.

**Figure 2:**
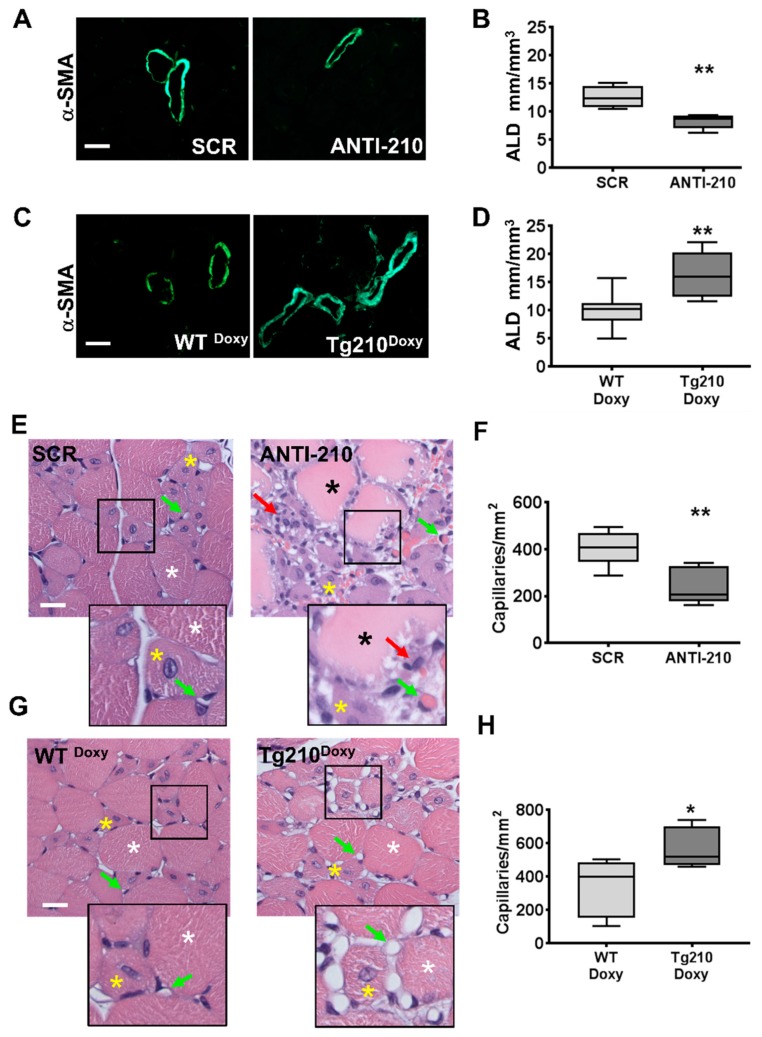
MiR-210 modulates arteriolar length density and capillary density. (**A**,**C**) Representative α-SMA immunofluorescence staining of (**A**) SCR and ANTI-210 and (**C**) WT^Doxy^ and Tg210^Doxy^ gastrocnemius muscle sections, 7 days after ischemia. Magnification is set at 400×, and the calibration bar is 20 µm. (**B**,**D**) Box plots show quantification of Arteriolar length density (ALD) (lumen minor diameter range: 4–10.99 µm) in SCR and ANTI-210 muscles (**B**: *n* = 5; test *t* ** *p* = 0.005) and in WT^Doxy^ and Tg210^Doxy^ mice (**D**: *n* = 6–10; test *t* ** *p* < 0.005). (**E**,**G**) Representative hematoxilin/eosine stained sections of ischemic gastrocnemius muscles of (**E**) SCR and ANTI-210, (**G**) WT^Doxy^ and Tg210^Doxy^ at day 7 after ischemia. Magnification is set at 400×, and the calibration bar is 20 µm. Green arrows indicate capillaries (that may contain an erythrocyte); red arrows indicate inflammatory infiltrating cells; white asterisks indicate healthy myofibers; yellow asterisks indicate regenerating myofibers; black asterisks indicate damaged myofibers. The insets show tissue structure at a higher magnification. F and H: Box plots show quantification of capillaries/mm^2^ in SCR and ANTI-210 muscles (**F**: *n* = 6, test *t* ** *p* = 0.007) and in WT^Doxy^ and Tg210^Doxy^ muscles (**H**: *n* = 6–10, test *t* * *p* = 0.02).

Arteriolar length density (ALD) was assessed on α-smooth muscle actin (α-SMA) stained sections 7 days after ischemia (Figure 2A–D). It was found that when miR-210 was blocked, ALD (4–10.99 µm) was lower compared to SCR controls (Figure 2A,B). Conversely, in Tg210^Doxy^ mice, ALD was significantly increased compared to WT^Doxy^ controls (Figure 2C,D).

Capillary density was also evaluated in hematoxylin/eosin stained sections of gastrocnemius muscles 7 days after ischemia (Figure 2E–H). After miR-210 blocking, capillary density significantly decreased (Figure 2E,F). Interestingly, similar results were also observed at day 14 after ischemia (Supplementary Figure S5). Conversely, when Tg210^Doxy^ mice were analyzed, a significantly higher capillary density was observed compared with WT^Doxy^ controls (Figure 2G,H).

Of note, capillary density was not affected in non-ischemic controlateral muscles of Tg210^Doxy^ mice (wt = 537.9 ± 24.6, Tg210^Doxy^ = 546.6 ± 16.0 capillaries/mm^2^; *n* = 10/group, *p* = non significant).

Collectively, data of blood perfusion and histology show a functionally relevant role of miR-210 in the neo-vascularization process following acute ischemia.

### 2.4. The MiR-210 Impact on the Transcriptome Indicates Regulation of Vascular Regeneration Pathways

In order to understand the consequences of miR-210 inhibition on the ischemia response, the ensuing transcriptomic changes were investigated using microarray analysis in gastrocnemius muscles of ANTI-210 and SCR treated mice, 7 days after ischemia. Using bioinformatics techniques, data were analyzed, normalized and filtered, and differentially expressed genes were identified.

MiRNAs target multiple genes, displaying in many circumstances a small regulatory effect on each target, but yielding a significant biological impact affecting several components of the same pathway [28]. Thus, instead of focusing on a small number of miR-210 targets, we concentrated on gene-ontology enrichment-analysis of the differentially expressed genes to detect coordinated gene-level differential-expression in specific pathways (Supplementary Figure S6). The bioinformatics step was followed by qPCR validation of a subset of genes belonging to different functional categories. A general concordance between modulations measured by qPCR and microarray was observed, confirming the validity of the approach (Supplementary Figure S7). This analysis allowed us to identify several terms falling into the biological macro areas of angiogenesis and blood vessel development, as well as related categories, such as cell adhesion, migration and proliferation. These findings are in agreement with the identified positive role played by miR-210 in the neo-vascularization process.

Many terms were also related to metabolism and mitochondrial organization, in keeping with the role of miR-210 in the regulation of oxidative phosphorylation [5,14].

These results indicate that miR-210 has a significant impact on the ischemic tissue transcriptome.

### 2.5. The miR-210 Expression Enhances Cardiac Function and Improves Vascular Regeneration after Acute Myocardial Infarction

To further corroborate the role of miR-210 in tissue repair after ischemic injury, we took advantage of a mouse model of acute myocardial infarction (MI). To this aim, Tg-210 female mice and WT littermate underwent coronary artery ligation. Sham-operated mice were used as controls. Three days later, the efficiency of the surgery was evaluated by echocardiography (Supplementary Figure S8A). A similar decrease in left ventricular fractional shortening was observed in WT^Doxy^ and Tg210^Doxy^ MI mice compared to sham controls, indicating that MI of comparable severity was induced in both groups (Supplementary Figure S9). Next, doxycycline administration started and was maintained until the end of the experiment (Supplementary Figure S8A). As observed in the skeletal muscle, doxycycline administration significantly increased miR-210 level in the heart of Tg210^Doxy^ mice (Supplementary Figure S8B).

**Figure 3:**
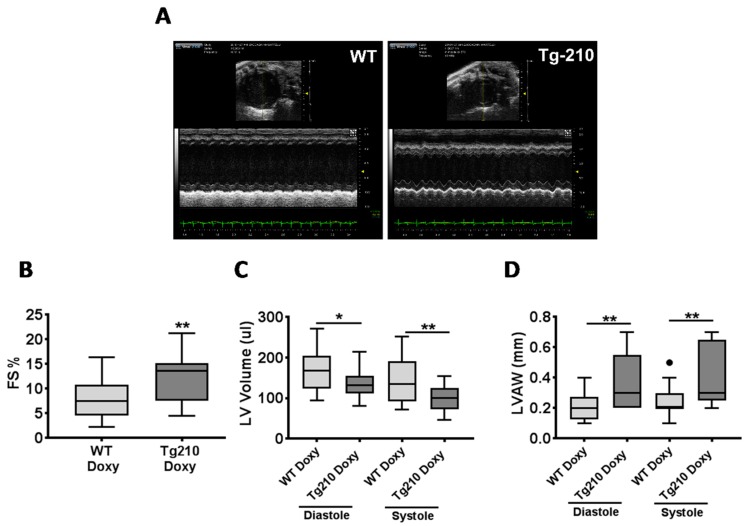
MiR-210 expression enhances cardiac function after myocardial infarction (MI). M-mode echocardiography is shown. (**A**) Representative parasternal short axis views of the LV, 1 month after MI in WT^Doxy^ e Tg210^Doxy^ mice. The box plots represent (**B**) The percentage of fractional shortening (FS %) (*n* = 18–20 ** *p* = 0.008); (**C**) The left ventricular volume (LV volume) (*n* = 18–20; * *p* = 0.02, ** *p* = 0.008); (**D**) The left ventricular anterior wall (LVAW) (*n* = 18–20; ** *p* ≤ 0.006), of WT^Doxy^ and Tg210^Doxy^ hearts. Dark spot indicates an outlier.

When cardiac remodeling and function were evaluated by echocardiography at 1 month after MI (Figure 3A), fractional shortening was significantly higher (Figure 3B), while left ventricular volume was significantly lower (Figure 3C) in Tg210^Doxy^ mice compared to WT^Doxy^. In addition, left ventricular anterior wall was significantly thicker in Tg210^Doxy^ mice compared to WT^Doxy^ (Figure 3D).

Masson trichrome staining of heart sections evaluated at the level of papillary muscles 30 days after MI confirmed echocardiographic results (Supplementary Figure S10).

**Figure 4:**
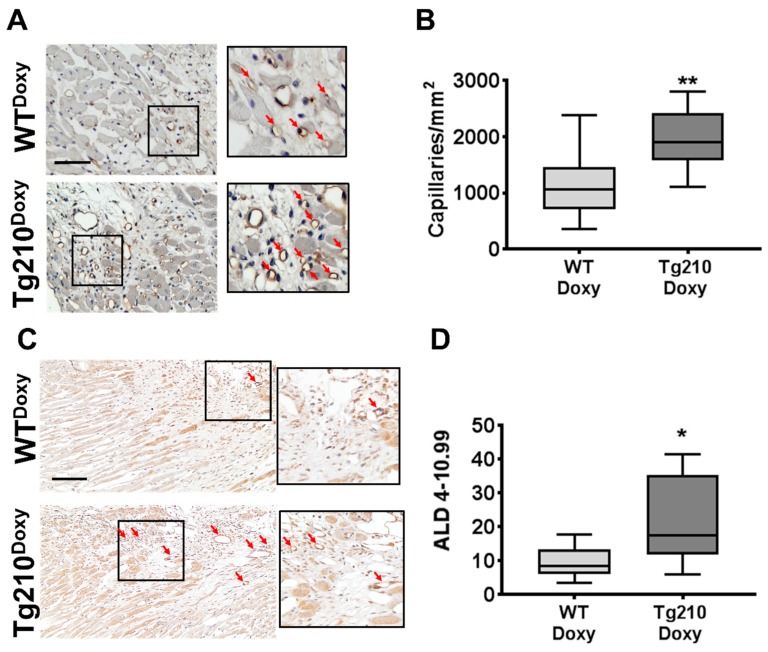
MiR-210 expression improves capillary density and arteriolar length density after MI. Morphometric analysis of capillaries and arteries after MI is shown. (**A**) Representative images of the border zone of the heart 1 month after MI, stained by Lectin immunohistochemistry. The magnification is shown at 400×, and the calibration bar at 50 µm. The inset shows a higher magnification; red arrows indicate capillaries. (**B**) The box plot shows quantification of capillaries/mm^2^ carried out in the peri-infarct region of WT^Doxy^ and Tg210^Doxy^ hearts (*n* = 9 ** *p* < 0.009). (**C**) Representative images of the border zone of the heart 1 month after MI, stained by αSMA immunohistochemistry. As expected in failing hearts, some αSMA expression was observed also in cardiomyocytes. Magnification is shown at 200×, and the calibration bar at 100 µm. The inset shows a higher magnification; red arrows indicate arterioles. (**D**) The box plot shows quantification of ALD carried out in the peri-infarct region and in the scar of WT^Doxy^ and Tg210^Doxy^ hearts (*n* = 8–9 * *p* < 0.01).

The morphometric analysis of the vasculature was assessed using immunohistochemistry for Lectin as a marker of endothelial cells (Figure 4A), and αSMA as a marker of arteries (Figure 4C). Both capillary density and ALD (4–10.99 µm) in the peri-infarct region of LV were significantly enhanced in Tg210^Doxy^ mice compared to WT^Doxy^ (Figure 4B,D, respectively).

Taken together, these results show that miR-210 expression improves cardiac contractility and leads to a more favorable left ventricular remodeling.

### 2.6. miR-210 Blocking Decreases Angiogenesis in Matrigel Plug Assays

To assess whether miR-210 can modulate blood vessel formation also in the absence of ischemic injury, we took advantage of the Matrigel plug, a well-established model of in vivo neovascularization. ANTI-210 or SCR oligonucleotides were administered 2 days before and 5 days after Matrigel plugs’ implantation and the angiogenic response were analyzed at day 7 (Supplementary Figure S11).

Matrigel plugs were formalin-fixed for histological analysis. Since RNA extraction from Matrigel is technically challenging, miR-210 inhibition was demonstrated in adductor muscles and in skin samples neighboring the plugs (Supplementary Figure S12).

**Figure 5:**
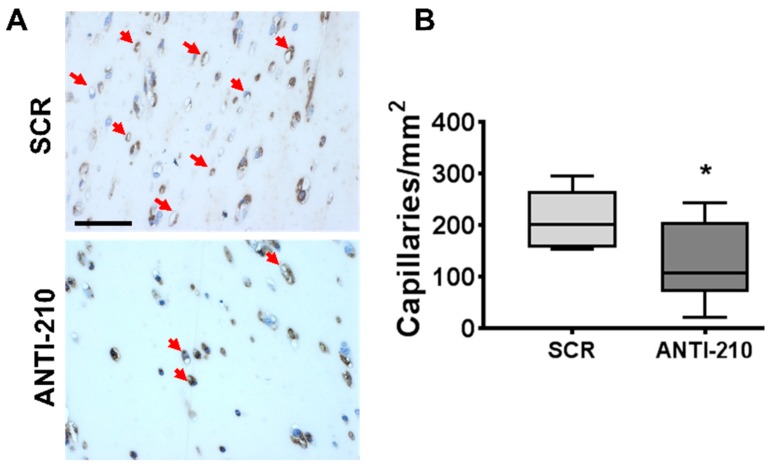
MiR-210 blocking decreases angiogenesis in Matrigel plugs in vivo. (**A**) Representative lectin staining of capillaries in Matrigel plugs sections 7 days post-implant. Magnification is shown at 400×; the calibration bar is at 50 µm. Red arrows indicate capillaries. (**B**) The box plot of capillary density quantification (*n* = 9–10, test *t*, * *p* = 0.03).

Angiogenesis was evaluated by lectin staining of endothelial cells in Matrigel plug histological sections (Figure 5A). It was found that miR-210 blocking significantly decreased capillary density compared to SCR controls (Figure 5B).

Taken together, these results indicate that miR-210 plays a pro-angiogenic role in vivo also in the absence of ischemic injury.

## 3. Discussion

The restoration of tissue homeostasis after an event of acute peripheral ischemia requires complex multicellular processes carried out by muscle resident cells, such as endothelial cells, pericytes, smooth muscle cells, and satellite cells, as well as by infiltrating inflammatory cells [29,30]. These mechanisms are clearly insufficient in patients with peripheral artery disease and critical limb ischemia in particular, prompting the need for identifying molecular effectors capable of stimulating the vascular regeneration process [2,3,4].

In this study, we showed that miR-210 is a crucial player in the events prompting capillary and arteriole regeneration in a mouse model of critical ischemia. These results were generated with a robust experimental strategy using both loss- and gain-of-function models. This way, while all methods have caveats and limitations, by using independent techniques, i.e., complementary oligonucleotides for miR-210 inhibition and inducible transgenic mice for miR-210 overexpression, we obtained fully consistent, complementary results indicating a pro-angiogenic role of miR-210 upon ischemia. Indeed, we found that miR-210 expression was necessary and sufficient for the functional recovery of blood perfusion following ischemia. These data were supported by morphometric analysis of capillary and arteriolar densities.

In keeping with the data obtained in the limb ischemia model, miR-210 expression also increased angiogenesis in a model of myocardial infarction, preserving cardiac contractility and leading to a more favorable remodeling of the left ventricle. In spite of obvious differences, in both models, increased capillary and arteriolar densities were observed upon miR-210 expression, indicating that the vascular cells are highly responsive to miR-210 levels.

Of note, hindlimb and heart ischemia experiments were conducted in male and female mice, respectively, indicating that the pro-angiogenic function of miR-210 is not sex-dependent.

These findings are in agreement with studies on the pro-angiogenic role of miR-210 observed in vitro [15,31,32,33], as well as in miR-210 overexpression studies in mouse models of heart [20,21,22,23], limb [26], and brain ischemia [24,25,34]. Also concordant are studies showing that cord blood-derived cells expressing miR-210 display an increased potential in stimulating angiogenesis upon transplantation in ischemic tissues [31,35].

While assessing miR-210 function in the context of ischemia is of obvious physio-pathological relevance, we found that miR-210 expression was also necessary in the neo-angiogenic process stimulated by VEGF and FGF2 in Matrigel plugs, consistent with observations in non-ischemic adult mouse brains [36].

Of note, in the contralateral non-ischemic tissue of Tg210^Doxy^, capillary density was unaffected, indicating that an additional stimulus, represented by the ischemic damage or by the presence of pro-angiogenic factors, is necessary for miR-210 to stimulate angiogenesis.

In order to investigate the molecular mechanisms underpinning the pro-angiogenic function of miR-210, we analyzed the transcriptomics changes associated to miR-210 blocking upon ischemia, followed by gene ontology analysis of the deregulated pathways. We speculated that this analysis could be particularly informative since each miRNA can target multiple mRNAs and its effect can be mediated not only by subtle effects on mRNA stability, but also by translation inhibition [37]. Accordingly, in our model, no previously validated miR-210 targets [6,7] were found to be deregulated significantly.

The validity of this approach was supported by the finding that many gene ontology categories were related to angiogenesis and blood vessel development, as well as related categories, such as cell adhesion, migration and proliferation, stress and inflammation responses. Moreover, many terms were related to metabolism and mitochondrial organization, consistent with the role of miR-210 in the regulation of oxidative phosphorylation [5,7,14].

Indeed, the cellular mechanism driving the pro-angiogenic function of miR-210 in peripheral ischemia are likely complex, possibly involving multiple cell types and molecular mechanisms. Moreover, the role played by miR-210 might be highly context-dependent [6,7]. In different experimental settings, miR-210 has been shown to stimulate the secretion of pro-angiogenic factors, such as VEGF and FGF2 [23,25,36,38,39,40], which may play a role in the vascular regeneration process also in the context of hindlimb ischemia. Finally, it is worth noting that miR-210 can be secreted into exosomes and, in this way, transferred from cell to cell, further complicating the scenario [20,26,41].

In conclusion, the present study demonstrates that miR-210 plays a pivotal role in post-ischemic neovascularization, suggesting that it could represent a potential therapeutic target in peripheral artery disease. Indeed, miR-210 appears to be a particularly promising one, since its beneficial action is not limited to the early period of ischemic damage when its anti-apoptotic, pro-survival functions can be exploited [6,10,18,19], but also to the following regenerative phases, when its pro-angiogenic action may be harnessed.

## 4. Material and Methods

An expanded version of materials and methods is shown in the Supplementary material section.

### 4.1. Mouse Models

All experimental procedures complied with the Guidelines of the Italian National Institutes of Health and with the *Guide for the Care and Use of Laboratory Animals* (Institute of Laboratory Animal Resources, National Academy of Sciences, Bethesda, MD, USA) and were approved by the institutional Animal Care and Use Committee (IACUC 666, approval date 02/19/2015, authorization 96/2015-PR and IACUC 709, approval date 08/31/2015, authorization 221/2015-PR). Down modulation of miR-210 was carried out by intraperitoneal injection of 12 mg/kg LNA-oligonucleotides against miR-210 (ANTI-210) or a scrambled control sequence (SCR) (In vivo LNAmicroRNA Inhibitors; Exiqon Vedbaek, Denmark) in C57BL/6N male mice (8–12 weeks old, Charles River laboratories, Calco (Lecco), Italy), as previously described [10]. Doxycycline inducible transgenic C57BL/6NTac-*Gt(ROSA)26Sor^tm3720(Mir210)Tac^* male mice (Tg210) and Wild Type littermates (WT) were previously described and characterized [18]. When miR-210 expression was induced, WT and Tg210 mice were fed with food containing doxycycline (WT^Doxy^ and Tg210^Doxy^) (Mucedola, Settimo Milanese (MI) Italy, NFM18 diet added with Doxycycline hyclate 2000 mg/kg).

Mice were anesthetized with an intraperitoneal injection of 10 mg/kg xylazine (Intervet Farmaceutici, Milan, Italy) and 100 mg/kg ketamine (Ketavet 100; Intervet Farmaceutici, Milan, Italy). Acetaminophen 1mg/mL was administrated in drinking water as an analgesic drug. Before samples harvesting, mice underwent euthanasia by anesthetic overdosing.

Acute hindlimb ischemia was induced by removing the femoral artery, as previously described [27]. Next, miR-210 up- or down-modulatory treatments were administered. While ANTI-210 oligos were injected as a bolus at 5 days after ischemia, doxycycline was assumed by the mice in the food. Thus, food containing doxycycline was provided at day 4 after ischemia to give sufficient time to elicit the desired miR-210 induction. Calf perfusion measurements were carried out by VEVO 2100 Ultrasound (FUJIFILM Visualsonics Inc., Toronto, ON, Canada) in power Doppler mode under general anesthesia by 1.5–2% isoflurane (Iso-Vet, Piramal Critical Care, West Drayton, UK ). Residual calf perfusion was expressed as vascularity ratio (left ischemic/right non-ischemic) [18].

Myocardial infarction was induced by coronary artery ligation in 8–12 weeks old Tg210, female mice and WT littermate under anesthesia and mechanically ventilated. Transthoracic echocardiography was performed using a high-performance ultrasonographic Imaging System (Vevo 2100; FUJIFILM Visualsonics Inc., Toronto, ON, Canada). Two-dimensional short-axis images and M-mode tracings were recorded at the level of papillary muscles. From M-mode tracings, anatomical parameters in diastole and systole were obtained.

The in vivo angiogenic Matrigel assay was performed as previously described [42], using Matrigel™ Basement Membrane Matrix (CULTREX, Trevigen, Helgerman Court, Gaithersburg, MD 20877 USA) loaded with pro-angiogenic factors (200 ng/mL VEGF, 1 mg/mL FGF2 and 0.1 mg/mL Heparin).

### 4.2. Sample Preparations

For RNA extraction, muscles were snap-frozen in liquid nitrogen. For histological analysis, mice were perfused with PBS pH 7.5, followed by 10% buffered formalin, at 100 mm/Hg for 10 min [27]. Next, gastrocnemius muscles were harvested, fixed, and paraffin-embedded, and sections were prepared as previously described [27]. For Matrigel assay, matrigel plugs were dissected and processed for paraffin inclusion and sectioning.

### 4.3. Histology and Morphometric Analysis

Capillary density was measured by counting the number of capillary profiles in hematoxylin/eosine stained sections [10] of muscle. For the quantification of capillary density in MI and Matrigel plugs, Lycopersicon esculentum lectin (Vector Laboratories, Burlingame, CA, USA) stained sections [42] were used. Also, 30–40 random fields/section were evaluated at 1000× magnification for muscles and Matrigel plugs. Only for MI samples, the quantification of capillary density was carried out in the peri-infarct region of the heart in 11–16 random fields/section at 40X magnification.

Arterioles were labelled by alpha−smooth muscle actin (α−SMA) antibody (Sigma-Aldrich, Saint Louis, MO, USA, #A5228) staining. Arterioles were visualized at 400× magnification and ALD was determined as previously described [27]. The quantification of ALD was carried out in the whole section of gastrocnemius muscles, while for MI, the peri-infarct region of the heart and the scar were analyzed. All quantifications were performed by 2 experienced histologists in blind.

### 4.4. miRNA and mRNA Quantification

Total RNA was extracted using TRIzol (Invitrogen, Life Technologies Corporation, Carlsbad, CA 92008 USA) and the TissueLyser system (Qiagen, Ambion Inc., Austin, TX, USA). The miRNA and mRNA levels were analyzed using the TaqMan qPCR assay (Applied Biosystems, Foster City, CA, USA) and the SYBR-GREEN qPCR method (Qiagen, Ambion Inc., Austin, TX, USA), respectively, and quantified with the Step-One plus real-time PCR System (Applied Biosystems, Foster City, CA, USA), as previously described [18]. Primers are listed in Table S1 (Supplementary methods). MiRNA and mRNA relative expression was calculated using the comparative Ct method (2–Delta Delta Ct) [43], and the expression values were normalized to miR-16 and RPL13 levels, respectively, both not modulated by ischemia or miR-210 [10,18].

### 4.5. Gene Expression and Bioinformatics Analysis

Gene expression profiles (*n* = 11/group) were measured using the TotalPrep RNA Amplification Kit (Ambion Inc., Austin, TX, USA) and the Illumina BeadChip Array MouseWG-6 v2 according to the manufacturer’s instructions. Next, GenomStudio’s bead summary probe level data were analyzed using Bioconductor. Sample intensities were quantile normalized and filtered for expression and probe quality using the beadarray R package [44]. Differential expression analysis was performed using the limma [45] R package [46], followed by ClueGO Enrichment analysis using the ClueGO app [47] for Cytoscape [48] pathway visualization program. At gene set level, dataset variability and subtle miRNA-dependent variations were addressed by setting only a cutoff to *p*-value < 0.05. ClueGO analysis was then performed by interrogating murine Gene Ontology Biological Process with a Benjamini-Hockberg correction for terms *p*-values, a term cutoff of FDR < 0.001, at least 50 supporting genes, and an ontology level cutoff between 1 and 4 to ensure terms related to major biological fluctuations.

### 4.6. Statistical Analysis

Variables were analyzed by Student’s *t* and ANOVA tests. All tests were performed 2-tailed and a *p*-value < 0.05 was considered statistically significant. Independent continuous variables were expressed in bar graphs as mean ± standard error and in box plots, representing data divided into quartiles. Outliers were identified by Tukey’s test. GraphPad Prism v.4.03 software (GraphPad Software Inc., San Diego, CA, USA) was used for statistical analysis.

## 5. Conclusions

miR-210 stimulates vascular regeneration and blood perfusion recovery in a mouse model of hindlimb ischemia.

miR-210 enhances cardiac function and improves cardiac remodeling after MI.

miR-210 stimulation of angiogenesis is independent of ischemia.

MiR-210 impacts on the transcriptome, regulating pathways related to vascular regeneration.

## Supplementary Materials

Supplementary materials can be found at https://www.mdpi.com/1422-0067/21/1/129/s1. Accession codes: Microarray-dataset are deposited in gene Expression Omnibus (GEO) database GSE142262.

## Abbreviations

Tg210	doxycycline inducible miR-210 transgenic mouse
ANTI-210	LNA-oligonucleotides against miR-210
SCR	scrambled control sequence
WT^Doxy^ and Tg210^Doxy^	WT and Tg210 mice fed with food containing doxycycline
α-SMA	alpha-Smooth muscle actin
ALD	arteriolar length density
MI	myocardial infarction
LV	left ventricle
