# Supplementary material for: Hypoxia-Induced miR-210 Is Necessary for Vascular Regeneration upon Acute Limb Ischemia
Source: Int J Mol Sci. 2019 Dec 24;21(1):129. doi: 10.3390/ijms21010129 (PMC6981725; doi:10.3390/ijms21010129)

Article

HYPOXIA-INDUCED MIR-210 IS NECESSARY FOR VASCULAR REGENERATION UPON ACUTE LIMB ISCHEMIA

Germana Zaccagnini[1*#], Biagina Maimone [1*], Paola Fuschi [1], Marialucia Longo [1], Daniel Da Silva [1], Matteo Carrara [1], Christine Voellenkle [1], Laura Perani [2], Antonio Esposito [2,3], Carlo Gaetano[4], Fabio Martelli [1#]

[1]Laboratory of Molecular Cardiology, IRCCS Policlinico San Donato, 20097 San Donato Milanese, Milan, Italy.

[2] Preclinical Imaging Facility, Experimental Imaging Center, San Raffaele Scientific Institute, 20132 Milan, Italy.

[3] Vita-Salute San Raffaele University, 20132 Milan, Italy.

[4] Laboratorio di Epigenetica, Istituti Clinici Scientifici Maugeri, via Maugeri 4, 27100 Pavia, Italy.

SUPPLEMENTAL DATA

Material and Methods

Mouse models

All experimental procedures complied with the Guidelines of the Italian National Institutes of Health and with the *Guide for the Care and Use of Laboratory Animals* (Institute of Laboratory Animal Resources, National Academy of Sciences, Bethesda, Md) and were approved by the institutional Animal Care and Use Committee (IACUC 666, approval date 02/19/2015, authorization 96/2015-PR and IACUC 709, approval date 08/31/2015, authorization 221/2015-PR).

Before all surgical and perfusion procedures, mice were anesthetized with an intraperitoneal injection of 10 mg/kg xylazine (Intervet Farmaceutici, Milan, Italy) and 100

mg/kg ketamine (Ketavet 100; Intervet Farmaceutici, Milan, Italy). One day before and 7 days after surgical procedure, acetaminophen 1mg/ml was administrated in drinking water as analgesic drug. Fresh solution was prepared every 3 days for maximum efficacy.

For ANTI-210 experiments, 8-12 weeks old C57BL/6N male mice (Charles River laboratories, Calco (Lecco), Italy) were used. For transgenic mouse experiments of hindlimb ischemia, 8-12 weeks old doxycycline inducible transgenic C57BL/6NTac-$Gt(ROSA)26Sor^{tm3720(Mir210)Tac}$ (Tg210, Taconic Artemis, Germany) male mice and Wild Type littermate (WT) were used. The generation of Tg210 mice has been previously described in detail[1]. Briefly, the miR-210 coding region was inserted into the ROSA26 locus by using a targeting strategy that allows doxycycline inducible overexpression of miR-210.

Acute hindlimb ischemia was induced by removing the femoral artery, as previously described[2].

Myocardial infarction was induced by coronary artery ligation in 8-12 weeks old Tg210, female mice and WT littermate under anesthesia and mechanically ventilated. Briefly, thoracotomy was performed via the third left-intercostal space and the left coronary artery was ligated. The chest was closed and the mice were allowed to recover. Sham-operated mice underwent a similar surgical procedure, except that the ligature around the coronary artery was not tied. Animals were euthanized 1 month after surgery.

All mice were housed in groups of three to five at 22°±2 C using a 12 h light-12 h dark cycle. Unless otherwise stated, animals were fed normal chow diet (SDS, irradiate VRF1). For miR-210 induction, WT and Tg210 mice were fed with pellets of food containing Doxycycline (Mucedola, Settimo Milanese (MI) Italy, NFM18 diet added with doxycycline hyclate 2000 mg/kg). Doxycycline was administrated to WT littermate too, in order to exclude side effects of the drug. The effectiveness of miR-210 induction was assessed by qPCR on quadriceps femoris muscles or liver samples of each mouse analyzed.

Inhibition of miR-210 *in vivo*

In vivo down modulation of miR-210 was carried out by intraperitoneal injection of LNA oligonucleotides against miR-210 (ANTI-210) or a scrambled control sequence (SCR) (In vivo LNAmicroRNA Inhibitors; Exiqon, Vedbaek, Denmark). The following 15mers LNA-

enhanced sequences with complete phosphothioate backbone were used: ANTI-210, GCTGTCACACGCACA; SCR, CGTCTAGCCACCTAG. WT mice underwent hind limb ischemia (day 0) and then they were randomized in two groups. After 5 days of ischemia, one group (ANTI-210) received one intraperitoneal injection of 12 mg/kg LNA-anti-miR-210 diluted in 200 µl of saline. The second group (SCR) received the same dose of scrambled sequence (SCR), as control. Both groups were sacrificed by overdose of anesthetic, 7 and 14 days after surgery. The effectiveness of ANTI-210 treatment was assessed by qPCR in each mouse on quadriceps femoris muscles or liver samples.

Ultrasonography and image analysis

Ultrasound imaging was performed using a high performance ultrasonographic Imaging System (Vevo 2100; FUJIFILM Visualsonics inc., Toronto, Ontario, Canada), with a 40 MHz linear probe (MD550; Vevo 2100; Fujifilm VisualSonics Inc., Toronto, Ontario, Canada).

Measurement of calf perfusion Calf perfusion measurement were performed as previously described[1]. Mice were maintained under general anesthesia obtained by 1.5–2% isoflurane (Iso-Vet, Piramal Critical Care, West Drayton, UK) vaporized in 100% oxygen (flow: 1l/min), in supine position. Body temperature was monitored with a rectal probe. During the acquisition the temperature of the animal was maintained between 37°±1 C and the administration of the anesthetic was adjusted to maintain the heart rate of the animal from 500 to 580 bpm. Measurement of perfusion were carried out by Ultrasound device VEVO 2100 (FUJIFILM Visualsonics inc., Toronto, Ontario, Canada) in ischemic and non-ischemic controlateral calves, using a 2100 transducer in power Doppler mode (transmit Power 100%; center frequency 32 MHz; gate 2; pulse repetition frequency; beam angle 0; Doppler gain 35 dB; dynamic range 35 dB), 7 days after ischemia. The calf percentage of vascularization (PV) is determined using the Vevo LAB analysis software (FIJIFILM VisualSonics Inc., Toronto, ON, Canada) that calculate the percentage of pixels which have a Power Doppler signal associated with them. Residual calf perfusion was expressed as vascularity ratio (left ischemic/right non ischemic).

Transthoracic echocardiography. Mice were initially anesthetized with 4% isoflurane with oxygen and maintained at 0,5-1% isoflurane with oxygen during imaging at a rate of 1liter/minute, administered via nose cone. Anesthetized mice were positioned supine on a heated imaging platform (THM150 MousePad part of the VisualSonics Vevo Integrated Rail System III) and legs taped to electrocardiograph (ECG) leads to monitor heart and respiration rates. Body temperature was monitored with a rectal probe. A depilatory cream (Veet, Reckitt Benkiser, Milan, Italy) was used to remove fur from the anterior thorax and prewarmed ultrasound gel (Aquasonic, Parker Laboratories Inc., Fairfield, NJ, USA) was used as a coupling agent between the ultrasound probe and the skin. The probe was held in position by a clamp mounted on the Vevo Rail System to avoid any compression of the thorax and any small movement of the probe. During the acquisition the temperature of the animal was maintained between 37°±1 C and the administration of the anesthetic was adjusted to maintain the heart rate of the animal from 500 to 580 bpm.

All image acquisitions and offline measurements were conducted by a single experienced operator who was blinded to animal groups. Frame rate of >200 frames per minute was maintained for all B-mode and M-mode images. M-mode short-axis images were recorded at the level of the papillary muscles and the LV was bisected to obtain an optimal M-mode selection. For the analysis of M –mode Images, the Vevo LAB analysis software (Fujifilm VisualSonics Inc., Toronto, ON, Canada) was used. Conventional echocardiographic measurements of the LV included Ejection Fraction (EF), Fractional shortening (FS), end-diastolic dimension (EDD), end-systolic dimension (ESD), anterior and posterior wall thickness, and mass.

Matrigel plug assay

The *in vivo* angiogenic Matrigel assay was performed as previously described[3]. Eight-ten weeks old C57Bl/6N male mice were treated with ANTI-210 or SCR 12 mg/kg 2 days before Matrigel injection (day -2). Next, 500 µl of Matrigel™ Basement Membrane Matrix (CULTREX, Trevigen, Helgerman Court, Gaithersburg, MD 20877 USA) were loaded with pro-angiogenic factors (200 ng/ml VEGF, 1 mg/ml FGF2 and 0.1 mg/ml Heparin) and injected subcutaneously along the abdominal midline. Seven days after the first injection

(day 5 after Matrigel implant), the mice received a second dose of LNA oligonucleotides. Two days later, at 7 days from Matrigel injection, mice were sacrificed and Matrigel plugs were carefully dissected, formalin fixed and processed for paraffin inclusion and sectioning. To analyze the efficacy of ANTI-210 treatment, skin and adductor muscles were snap-frozen for RNA extraction and miRNA quantification by qPCR.

Genotyping analysis

Genomic DNA was extracted from tail biopsies. Tails were incubated with proteinase K (LS004222, Worthington Biochemical Corporation, Lakewood, NJ, USA) 20mg/ml in DirectPCR® Lysis Reagent (102-T; Viagen Biotech, Inc. Los Angeles, CA, USA) over night (O.N) at 55°C and then they were incubated for 45 min at 85°C. 2 µl of lysates was used for PCR genotyping. PCR reaction was performed to identify the presence of the miR-210 coding region in the constitutive Knock-In 1 allele (656-bp fragment), using the following primers: forward primer 5′-CCTGCAATATTTGCATGTCG-3′ and reverse primer 5′-GTCCCTATTGGCGTTACTATGG-3′. The unmodified ROSA26 locus (299-bp fragment) was amplified as a control and to determine the zygosity of the locus, using the following primers: forward primer 5′-CTCTTCCCTCGTGATCTGCAACTCC-3′ and reverse primer 5′CATGTCTTTAATCTACCTCGATGG-3′. PCR conditions were as follows: pre-denaturation at 95°C for 5 min, followed by denaturation at 95°C for 30 s, primer annealing at 60°C for 30 s, and extension at 72°C for 1 min (35 cycles), and finally an additional extension at 72°C for 10 min[1]. Genomic DNA samples from founder TG-210 mice and from WT mice were used as positive and negative controls, respectively, in each PCR reaction. The reaction was analyzed on 1.5% agarose gels containing ethidium bromide.

Sample preparation

For RNA extraction, muscles were snap frozen in liquid nitrogen. For histological analysis of ischemic muscles, mice underwent euthanasia by overdose of anaesthesia and were perfused with PBS pH 7.5, followed by 10% buffered formalin, at 100 mm/Hg for 10 min[2]. The perfusion was carried out *via* left ventricle for gastrocnemius muscle harvesting and *via* abdominal aorta for heart harvesting. Next, samples were harvested, fixed and paraffin

embedded. For Matrigel assay, Matrigel plugs were carefully dissected and processed for paraffin inclusion and sectioning.

Tables

Table S1

The primers listed below were used for qPCR analysis:

	Forward	Reverse
Col1a1	ACAGTCGCTTCACCTACAGC	GGGTGGAGGGAGTTTACACG
Fn1	TCATGTTCCGGGCCTCAATC	ATGGCGTAATGGGAAACCGT
Col3a1	TGGCAACCCTGGAATAGCTG	CTGGCTCCTGGTTTTCCACT
Ap1b1	TGGACATGCTTCTGGTGTGA	GCAGGATCAGACATCCCCA
Ap1m1	AGCCCTTGACCACATCAGTG	GAGGCCTTGTACAGTGAGGG
Egf	ACTGGACGGTTTGCCTCTTT	GCGTTCCTGAGAGTGAGCTA
Mmp2	TGTATGCCCTTCGCTCGTTT	AGTGAGGAGGGGAACCATCA
MfsD7b	CACCTGTGTCTGCCTTCATC	ATAGGTGCCATTCCAGTTGC
Fgf1	ACCATCCGGGTATTTGCTCA	GGTGTGACTGACCGTTGAGG
Lox	GGACCGTGGTCTAGCTTTCC	GCTAGGACGTTGGGTAGCTG
Cited2	GGCAACATGAATGCCACGAG	TTGGGGTTGCAATCTCGGAA
Fzr1	GCATTGCCACCCTTTGTCTG	GGCAGTGGAGATGGGGTATG
Mdh1	AGCCCAATCAATTTCAGAGGGA	TCTGCAACCTTCTTACAGCTAA
Suclg2	GCTTCCAGAAGGGTACAGCTAA	GTACTGGAATTAGGGGCCAGTG

Table S1. Sequences of qPCR primers

Figure legends

Figure S1

MiR-210 induction in C57BL/6N mice following ischemia. The bar graph shows miR-210 levels measured by qPCR in ischemic gastrocnemius muscles, expressed as fold induction versus the non-ischemic controlateral muscles at different time points of hindlimb ischemia

(n=3-4; Two way Anova multiple comparison, non-ischemic versus ischemic *p=0.02; **P≤0.004, #P<0.0001).

Figure S2

Time course of capillary density after hindlimb ischemia in C57BL/6N mice. Capillary density was quantified in hematoxylin/eosin stained sections of ischemic gastrocnemius muscles compared to non-ischemic muscles at different times of ischemia. Box plot shows quantification of capillary density (n=4-6; Anova multiple comparison *P=0.01, **P=0.001; #P=0.0001).

Figure S3

Schematic representation of loss- and gain-of function experiments. A. Loss of function: schematic representation of miR-210 blocking in hindlimb ischemia. WT mice underwent hindlimb ischemia (day 0) and, at day 5, were randomized into two groups for further treatments. In one of the groups, miR-210 function was blocked by systemic administration of ANTI-210; the second group received a SCR sequence and was used as control. In both groups, the angiogenic response was analyzed 2 and 7 days later (at 7 and 14 days of ischemia respectively) by power Doppler and histological analysis. **B.** Gain of function: schematic representation of miR-210 overexpression. WT and Tg210 untreated mice (UT) underwent surgery at time 0. After 4 days and until the end of the study, both groups were fed with pellets of food containing doxycycline to induce miR-210 over-expression ($Tg210^{Doxy}$) or as control (WT^{Doxy}). The angiogenic response was analyzed 3 days later (at 7 days of ischemia) by power Doppler and histological analysis.

Figure S4

Effective miR-210 inhibition and overexpression *in vivo*. A. The bar graph shows miR-210 levels measured in ischemic quadriceps femoris muscles by qPCR and expressed as fold change versus the SCR controls, after 2 or 7 days of treatment (n=6-8, Two way Anova #P<0.0001). **B.** The bar graph shows miR-210 levels measured in non-ischemic gastrocnemius muscles by qPCR and expressed as fold induction versus WT controls, after doxycycline administration (n=3-9; Anova multiple comparison #P=0.0001).

Figure S5

MiR-210 modulates capillary density at 14 days of ischemia. Box plot shows quantification of capillaries/mm^2 in SCR and ANTI-210 gastrocnemius muscle sections at day 14 of ischemia. (n=6; test T **p<0.001).

Figure S6

GO Biological Process terms enriched upon miR-210 blocking. Terms characterized by significant enrichment are shown as coloured circles, connected by edges when the terms are functionally related in the GO tree. For each cluster of terms, the most significant and possibly an additional biologically meaningful one are captioned. Circles are coloured based on their membership to a specific branch the GO tree. Multiple colours define terms that are part of more than one biological process. (SCR n=11, ANTI-210 n= 11, FDR <0.001).

Figure S7

Heat map of validated differentially expressed genes. The level of the indicated genes was measured in SCR and ANTI-210 ischemic gastrocnemius muscles by microarrays analysis 7 days after ischemia and was validated by qPCR, (n=11/group). The heat map shows average expression levels where green and red colours indicate down- or upregulation, respectively. A general concordance between modulations measured by microarray and qPCR was observed.

Figure S8

A. Schematic representation of myocardial infarction experiments. WT and Tg210 untreated mice (UT) underwent surgery at time 0. After 3 days mice received transthoracic echocardiography to assess cardiac function. Starting from day 3 and until the end of the study, both groups were fed with food containing doxycycline to induce miR-210 over-expression ($Tg210^{Doxy}$) or as control (WT^{Doxy}). At day 30, mice underwent a second echocardiography and were euthanized for samples harvesting and histological analysis. **B**. Box plot represents miR-210 fold change measured by qPCR in healthy hearts after 5 days of doxycycline treatment (N=5; **P=0.002).

Figure S9

Fractional shortening assessment in sham operated or infarcted mice at day 3. Box plot represents the percentage of fractional shortening (%FS) measured in sham operated mice at day 3 or 30 (N=9) and in MI mice at day 3 after surgery (N=18-20). A similar %FS decrease was observed in $Tg210^{Doxy}$ and WT^{Doxy} mice upon MI.

Figure S10

Masson trichrome staining of infarcted hearts.

Representative Masson trichrome staining of WT^{Doxy} and $Tg210^{Doxy}$ hearts 30 days after MI. Images were taken at the level of papillary muscles. Magnification 25x, calibration bar 1000 µm.

Figure S11

Schematic representation of Matrigel plug experimental plan. C57Bl/6N mice were divided into two groups and treated with ANTI-210 or SCR oligonucleotides (day -2). Matrigel loaded with VEGF and FGF pro-angiogenic factors was injected subcutaneously 2 days later (day 0). After 5 days, Matrigel plug implanted mice received a second dose of LNA-oligonucleotides (day 5). At day 7, mice were sacrificed and Matrigel plugs were processed for paraffin inclusion and sectioning. To analyze the efficacy of ANTI-210 treatment, skin and adductor muscles were snap-frozen for RNA extraction and miRNA quantification by qPCR.

Figure S12

ANTI-210 efficiently inhibits miR-210 in skin and adductor muscle of mice implanted with Matrigel. The bar graph shows miR-210 levels after miR-210 blocking, measured by qPCR in skin and in adductor muscles and expressed as fold induction versus SCR samples (n=9-10; #P=0.0001).

References

1. Zaccagnini G, Maimone B, Fuschi P, Maselli D, Spinetti G, Gaetano C, Martelli F. Overexpression of miR-210 and its significance in ischemic tissue damage. *Sci Rep.* 2017; 7: 9563-017-09763-4.

2. Zaccagnini G, Palmisano A, Canu T, Maimone B, Lo Russo FM, Ambrogi F, Gaetano C, De Cobelli F, Del Maschio A, Esposito A, Martelli F. Magnetic Resonance Imaging Allows the Evaluation of Tissue Damage and Regeneration in a Mouse Model of Critical Limb Ischemia. *PLoS One.* 2015; 10: e0142111.

3. Di Stefano V, Cencioni C, Zaccagnini G, Magenta A, Capogrossi MC, Martelli F. p66ShcA modulates oxidative stress and survival of endothelial progenitor cells in response to high glucose. *Cardiovasc Res.* 2009; 82: 421-429.

FIG. S1

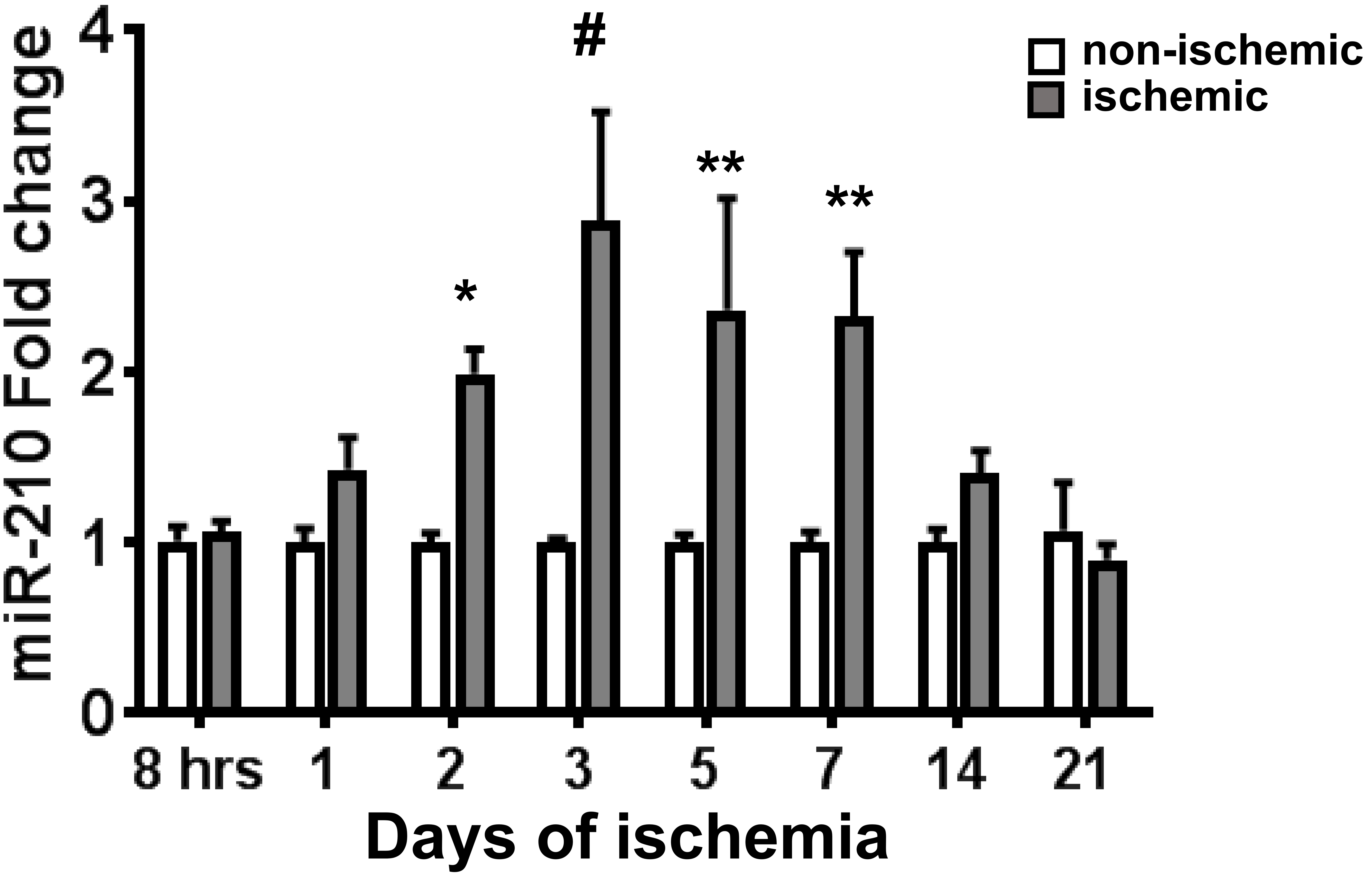

miR-210 Fold change
0
1
2
3
4
#
*
**
**
non-ischemic
ischemic
8 hrs
1
2
3
5
7
14
21
Days of ischemia

FIG. S2

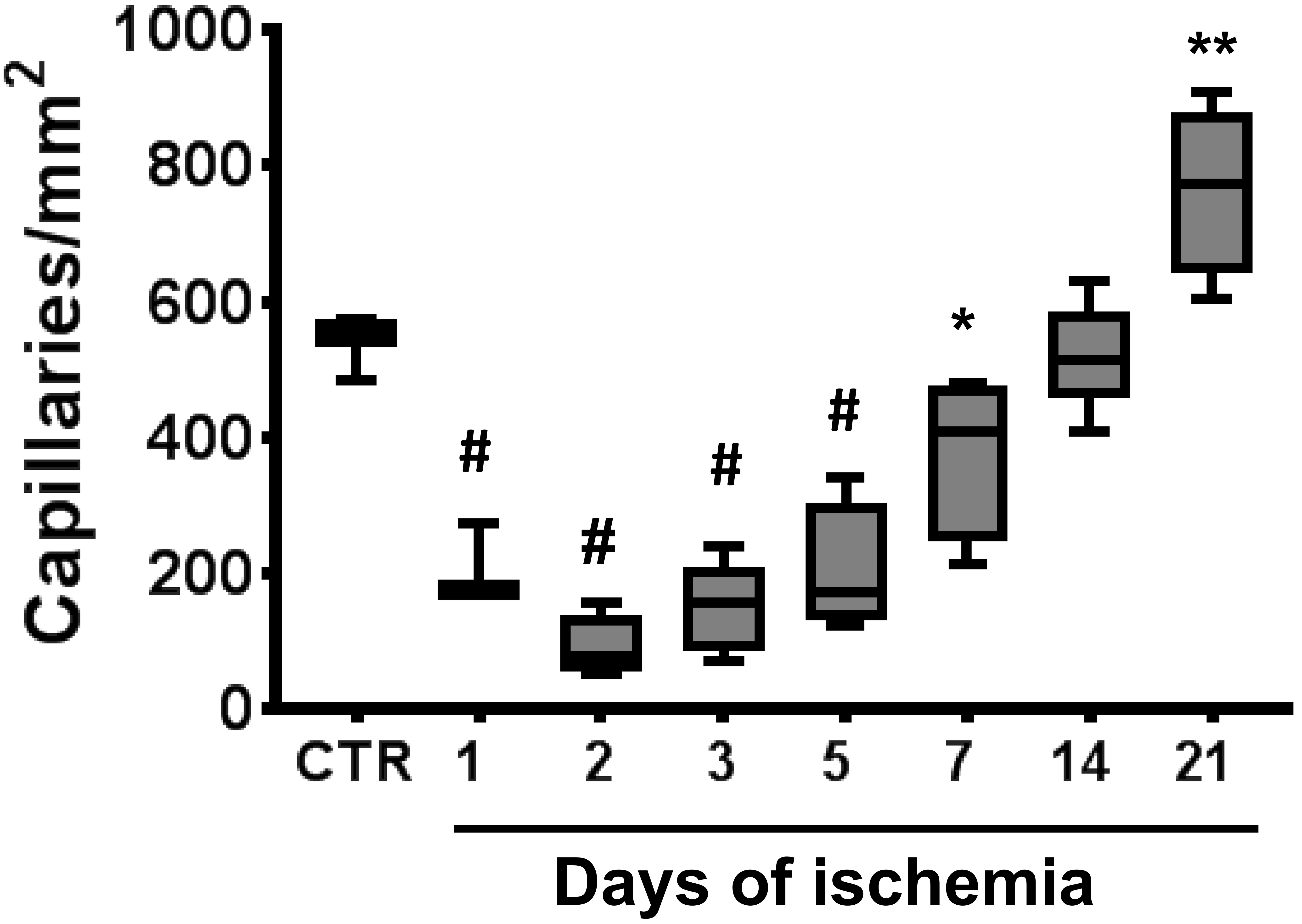
Capillaries/mm2
1000
800
600
400
200
0
#
#
#
#
*
**
CTR
1
2
3
5
7
14
21
Days of ischemia

FIG. S3

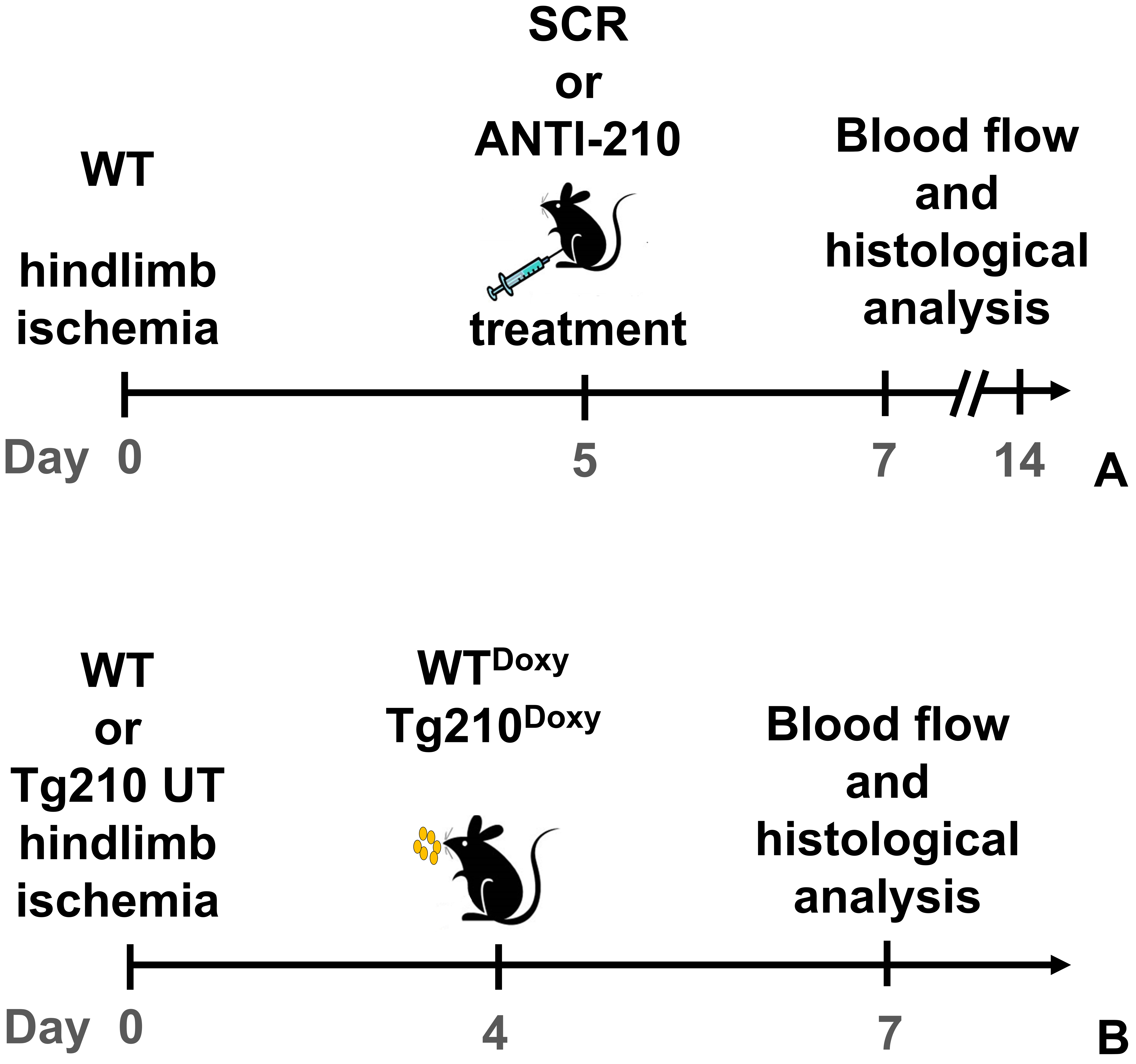
WT
hindlimb
ischemia
SCR
or
ANTI-210
treatment
Blood flow
and
histological
analysis
Day 0
5
7
14
A
WT
or
Tg210 UT
hindlimb
ischemia
WTDoxy
Tg210Doxy
Blood flow
and
histological
analysis
Day 0
4
7
B

FIG.S4

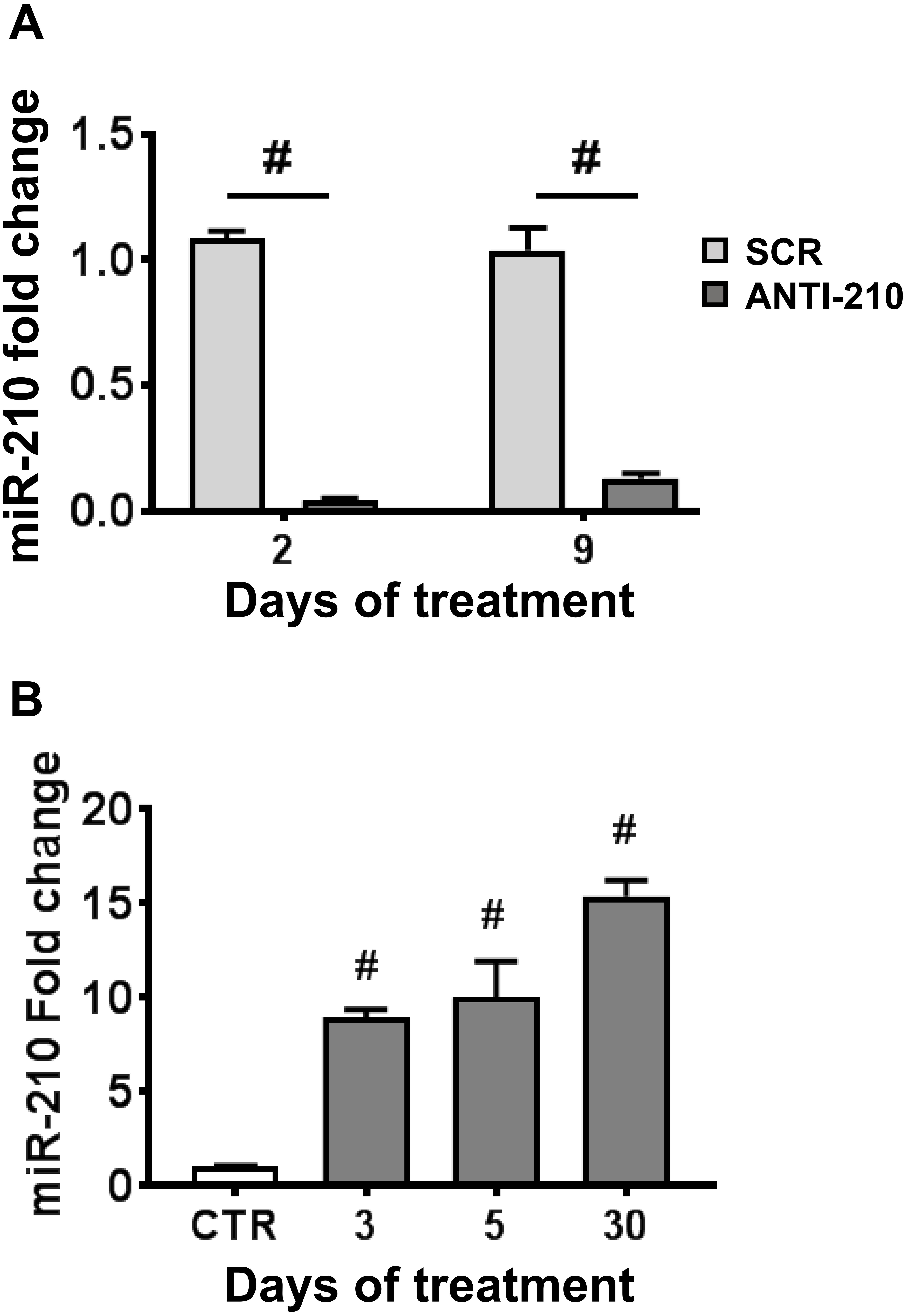
A
miR-210 fold change
1.5
1.0
0.5
0.0
#
#
SCR
ANTI-210
2
9
Days of treatment
B
miR-210 Fold change
20
15
10
5
0
#
#
#
CTR
3
5
30
Days of treatment

FIG.S5

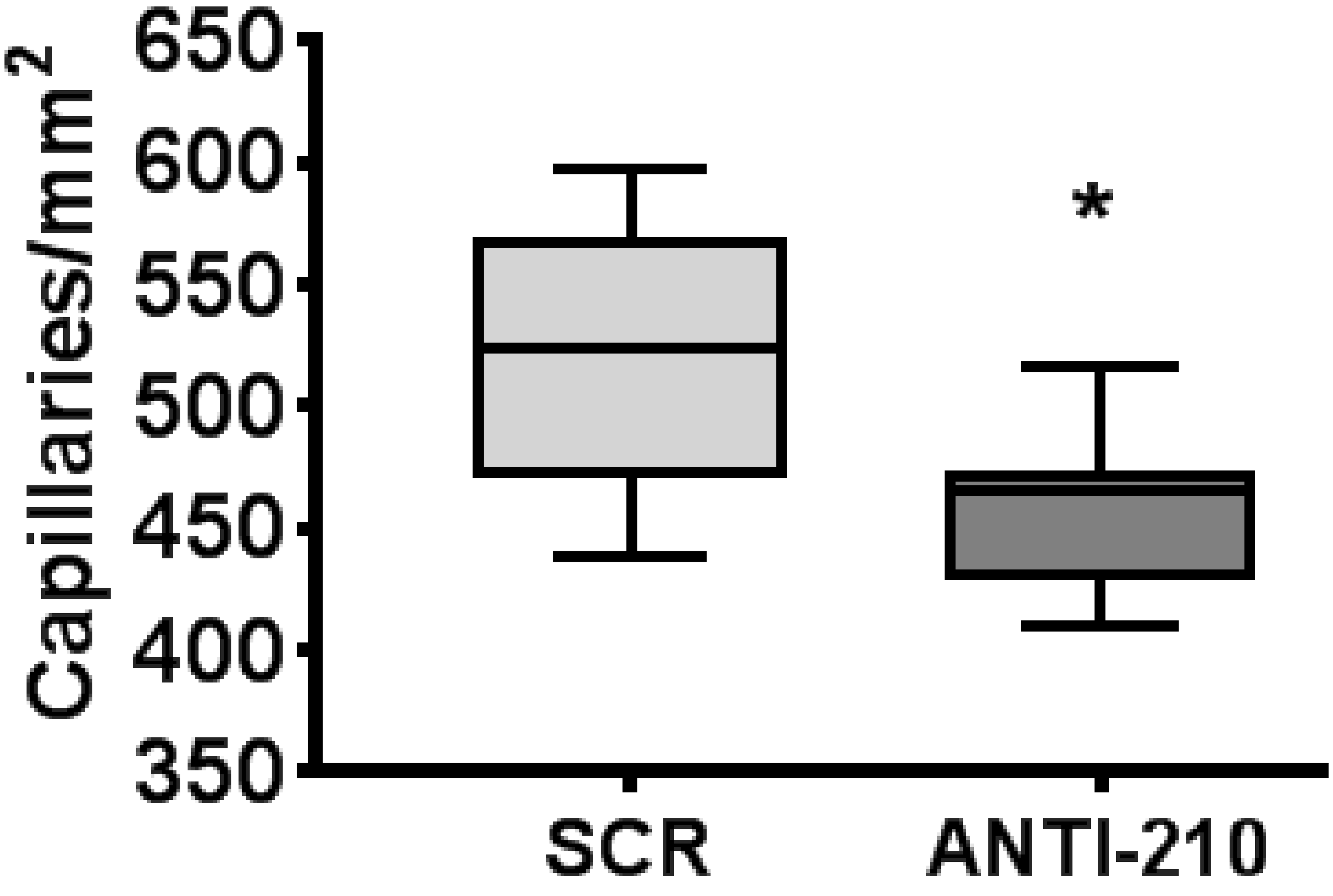

Figure S6

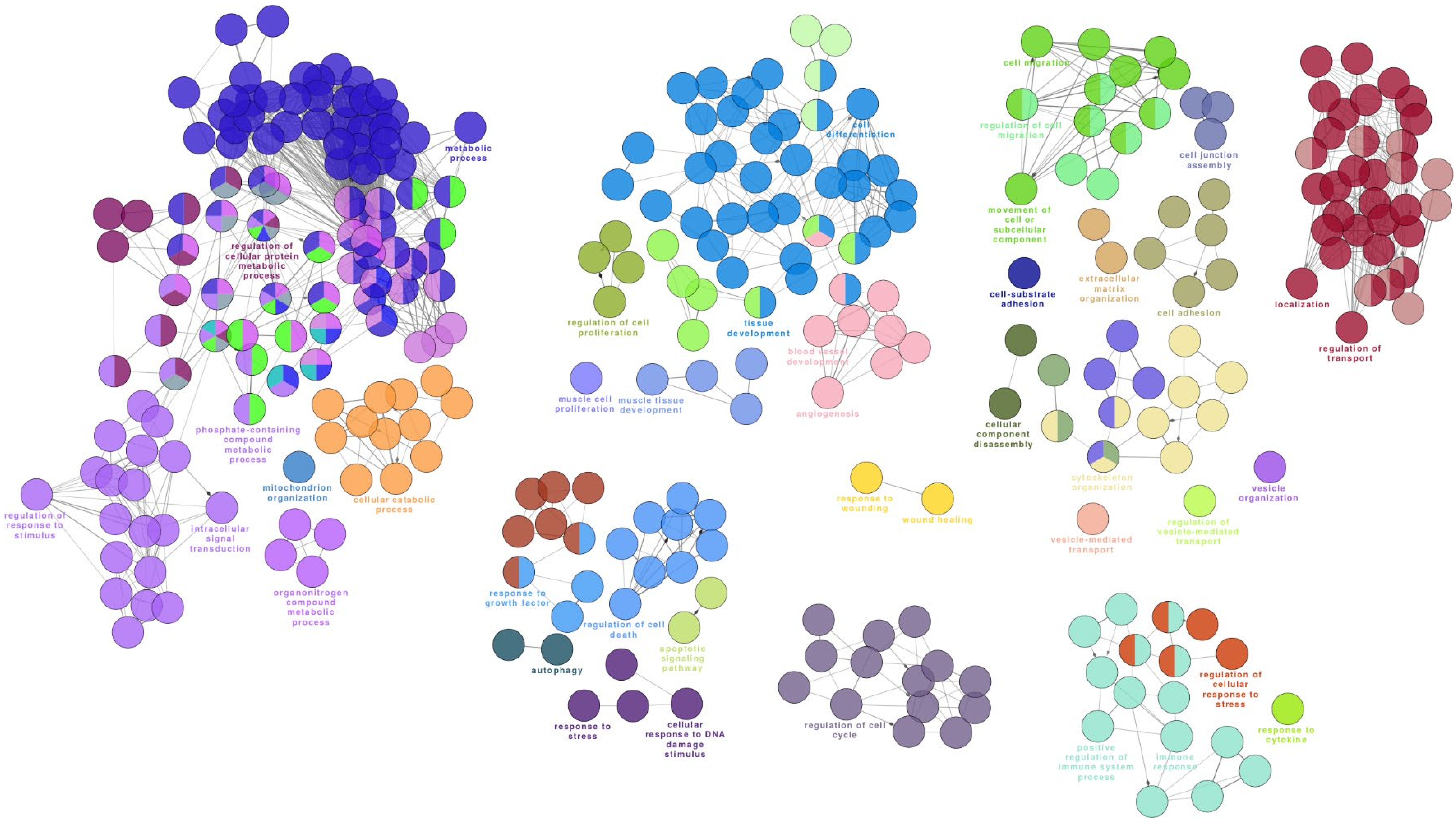

metabolic process
regulation of cellular protein metabolic process
phosphate-containing compound metabolic process
mitochondrion organization
cellular catabolic process
regulation of response to stimulus
intracellular signal transduction
organonitrogen compound metabolic process
cell differentiation
tissue development
regulation of cell proliferation
blood vessel development
angiogenesis
muscle cell proliferation
muscle tissue development
cell migration
regulation of cell migration
movement of cell or subcellular component
cell-substrate adhesion
extracellular matrix organization
cell adhesion
cell junction assembly
cellular component disassembly
cytoskeleton organization
localization
regulation of transport
vesicle organization
vesicle-mediated transport
regulation of vesicle-mediated transport
response to wounding
wound healing
response to growth factor
regulation of cell death
apoptotic signaling pathway
autophagy
response to stress
cellular response to DNA damage stimulus
regulation of cell cycle
regulation of cellular response to stress
response to cytokine
positive regulation of immune system process
immune response

FIG. S7

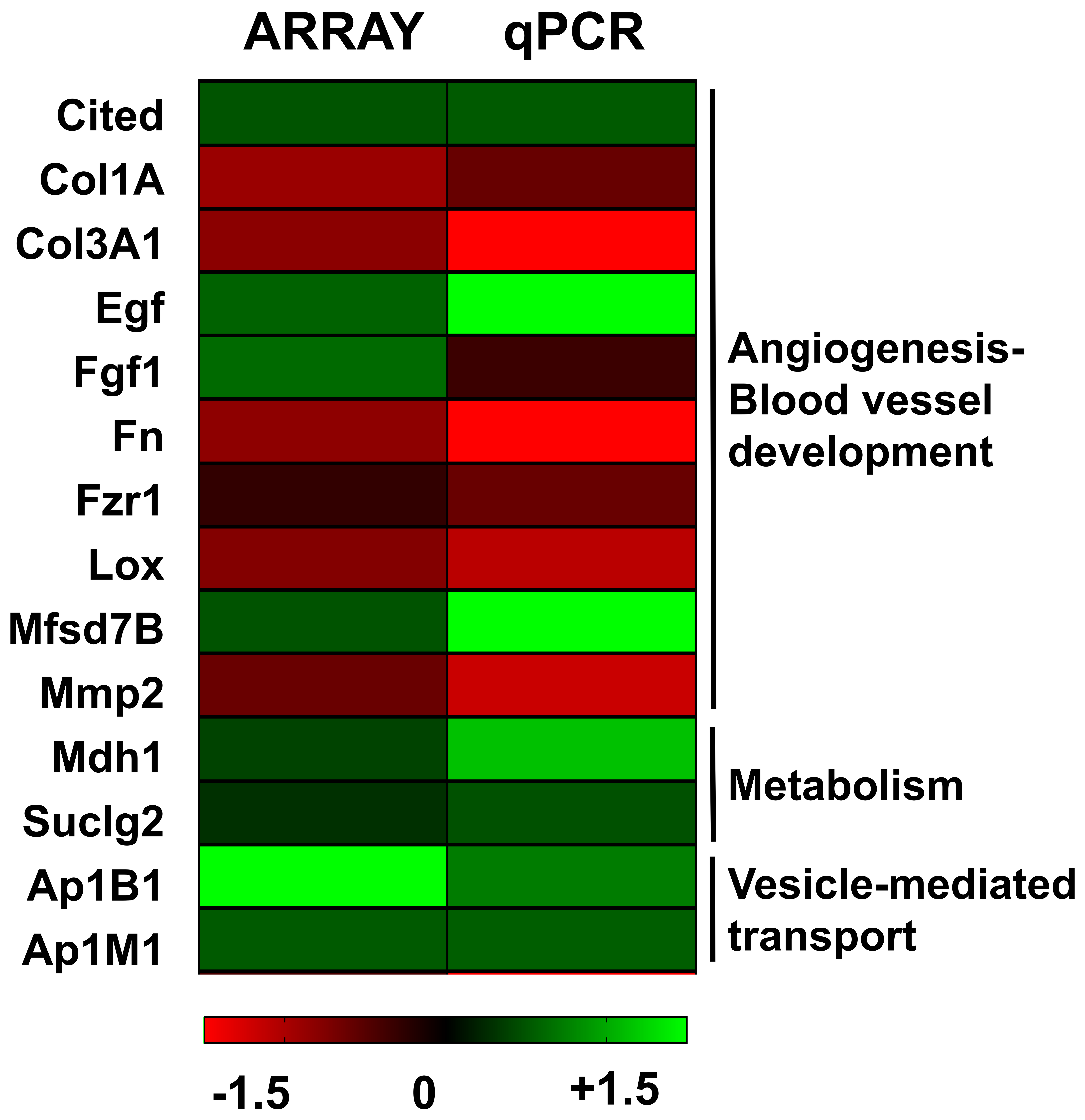
ARRAY
qPCR
Cited
Col1A
Col3A1
Egf
Fgf1
Fn
Fzr1
Lox
Mfsd7B
Mmp2
Mdh1
Suclg2
Ap1B1
Ap1M1
Angiogenesis-
Blood vessel
development
Metabolism
Vesicle-mediated
transport
-1.5
0
+1.5

FIG. S8

A

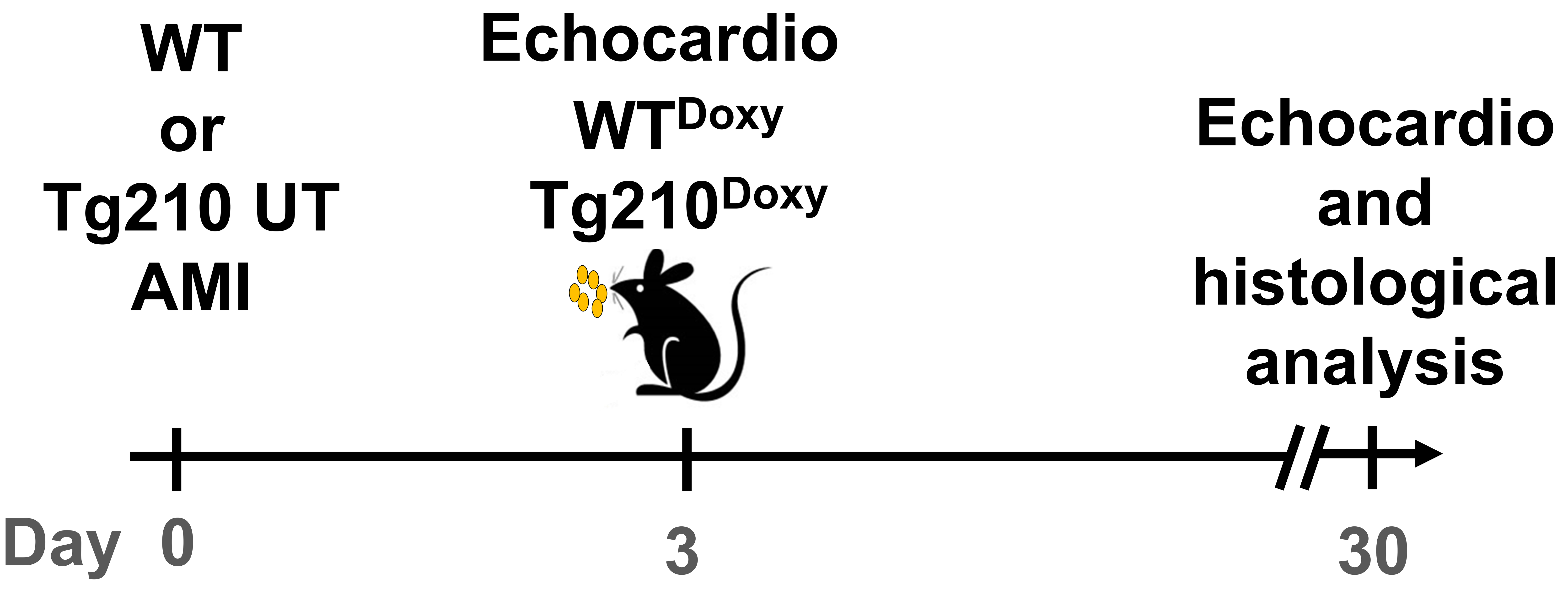
WT
or
Tg210 UT
AMI
Echocardio
WT^Doxy
Tg210^Doxy
Echocardio
and
histological
analysis
Day 0
3
30

B

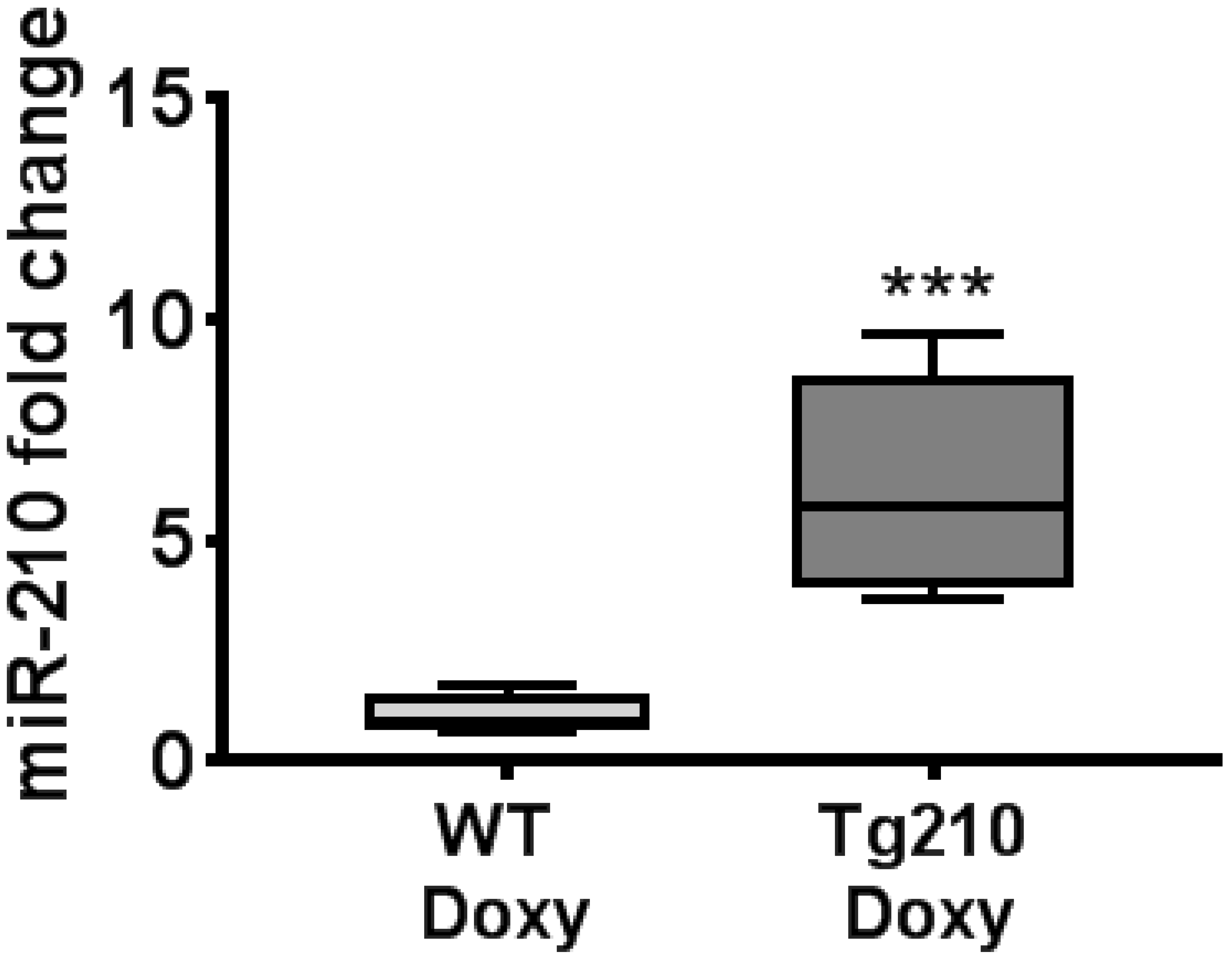
miR-210 fold change
15
10
5
0
***
WT
Doxy
Tg210
Doxy

FIG. S9

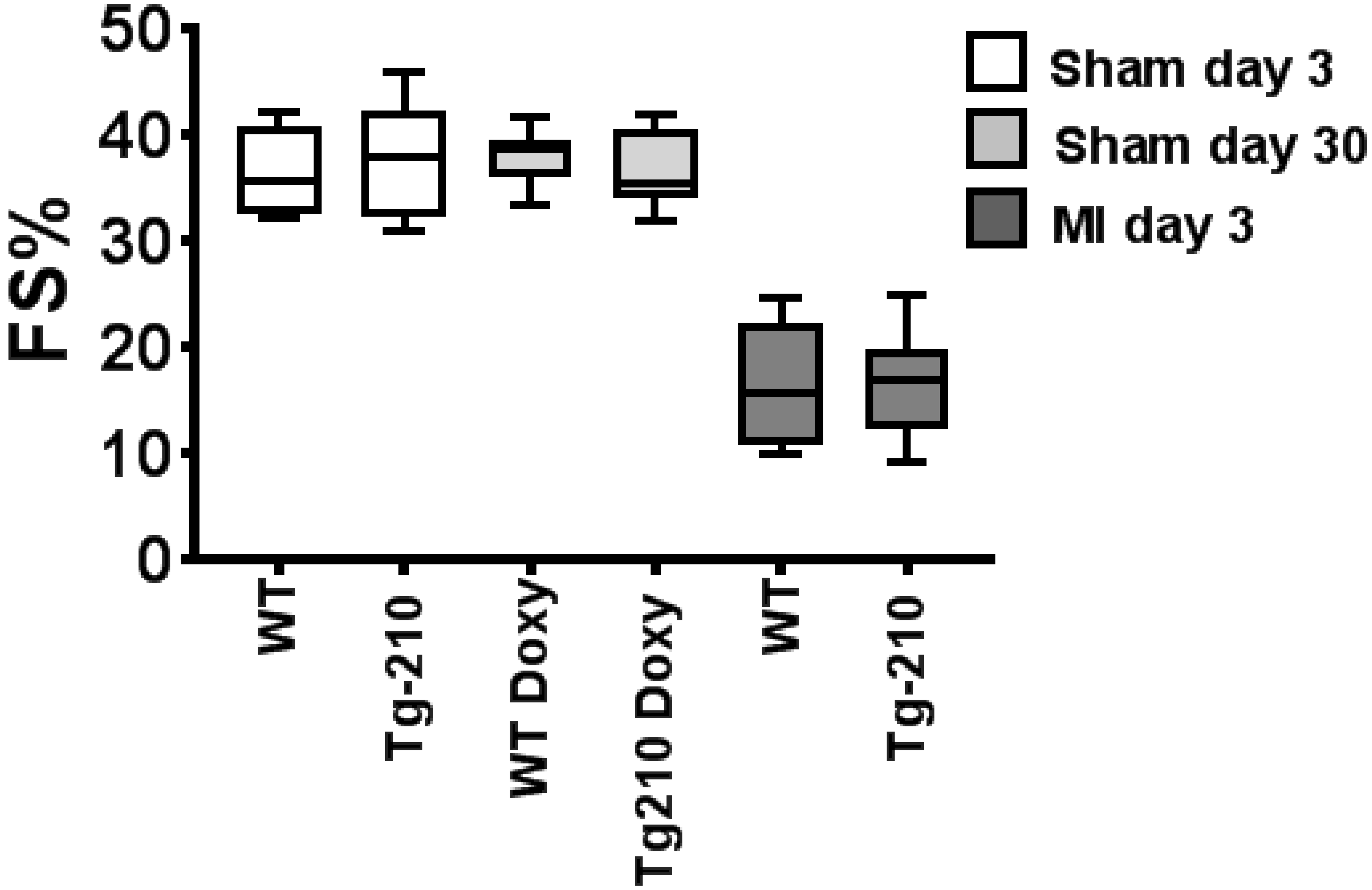

FS%
50
40
30
20
10
0
WT
Tg-210
WT Doxy
Tg210 Doxy
WT
Tg-210
Sham day 3
Sham day 30
MI day 3

Fig. S10

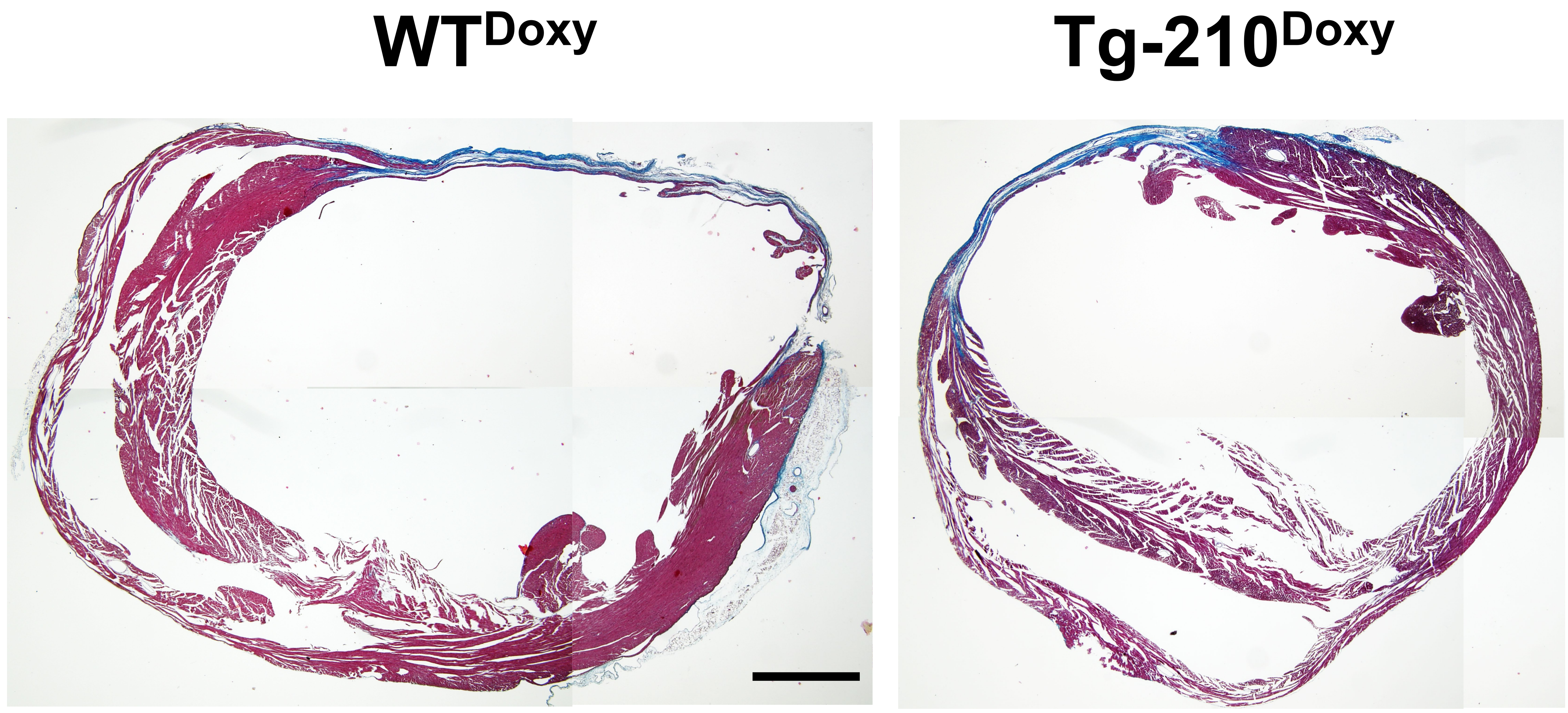

FIG. S11

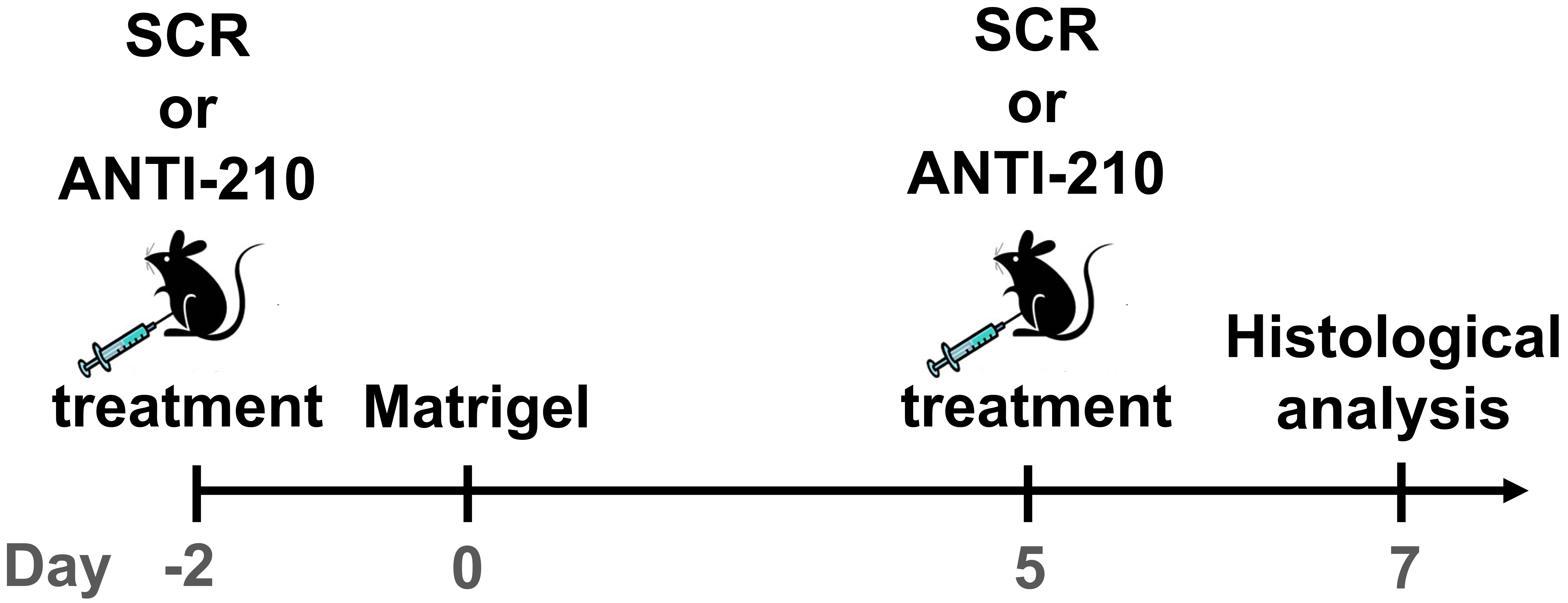
SCR
or
ANTI-210
treatment
Matrigel
SCR
or
ANTI-210
treatment
Histological
analysis
Day
-2
0
5
7

FIG.S12

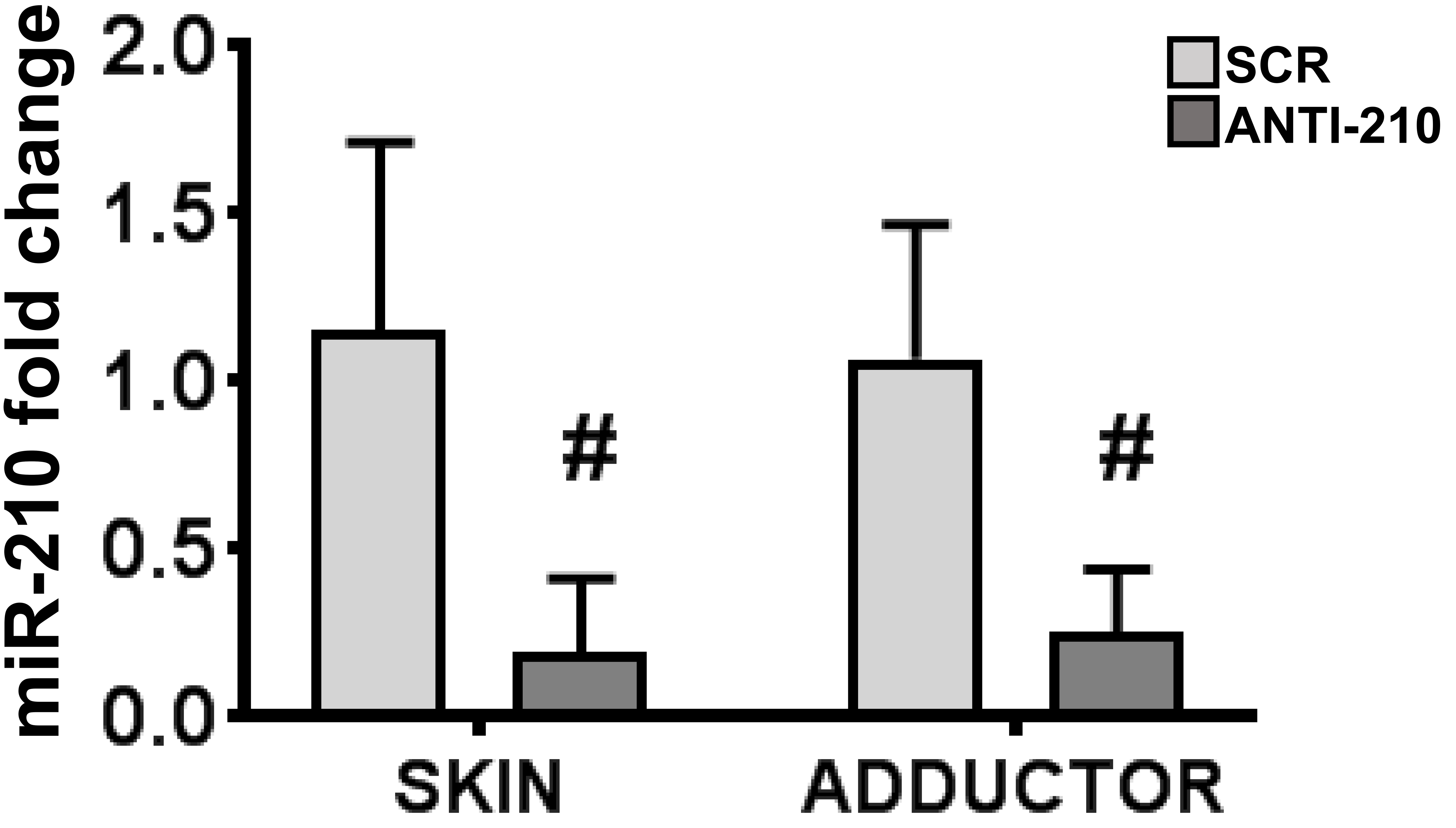

Supplement: Supplementary file 1 [file ijms-21-00129-s001.pdf]
